# NLRP3 deficiency decreases alcohol intake controlling anxiety-like behavior via modification of glutamatergic transmission in corticostriatal circuits

**DOI:** 10.1186/s12974-022-02666-w

**Published:** 2022-12-20

**Authors:** Ziyi Li, Olivia Ewi Vidjro, Gengni Guo, Yanfeng Du, Yao Zhou, Qian Xie, Jiaxin Li, Keqiang Gao, Li Zhou, Tengfei Ma

**Affiliations:** 1 Institute for Stem Cell and Neural Regeneration and Key Laboratory of Cardiovascular & Cerebrovascular Medicine, School of Pharmacy, Nanjing Medical University, 101 Longmian Street, Nanjing, 211166 Jiangsu China; 2 Department of Anesthesiology, Women’s Hospital of Nanjing Medical University, Nanjing Maternity and Child Health Care Hospital, 123 Tianfei Lane, Mochou Road, Nanjing, 210004 Jiangsu China; 3 Jiangsu Key Laboratory of Neurodegeneration, Nanjing Medical University, Nanjing, Jiangsu China; 4 Grade 2020 in Pharmacy, School of Pharmacy, Nanjing Medical University, Nanjing, Jiangsu China

**Keywords:** Alcohol, NLRP3 inflammasome, Synaptic plasticity, Glutamatergic transmission, Striatum

## Abstract

**Background:**

Alcohol use disorders result from repeated binge and chronic alcohol consumption followed by negative effects, such as anxiety, upon cessation. This process is associated with the activation of NLRP3 inflammasome-mediated responses. However, whether and how inhibition of the NLRP3 inflammasome alters alcohol intake and anxiety behavior remains unclear.

**Methods:**

A combination of drinking-in-the-dark and gavage was established in NLRP3-knockout and control mice. Behavior was assessed by open-field and elevated plus maze tests. Binge alcohol drinking was measured at 2 h and 4 h. A 2 h/4 h/24 h voluntary drinking was determined by a two-bottle choice paradigm. Western blotting and ELISA were applied to examine the levels of the NLRP3 inflammasome and- inflammatory factors, such as IL-1β and TNF-α. Nissl staining was used to measure neuronal injury. The electrophysiological method was used to determine glutamatergic transmission in corticostriatal circuits. In vivo optogenetic LTP and LTD were applied to control the function of corticostriatal circuits on the behavior of mice. MCC950 was used to antagonize the NLRP3 inflammasome.

**Results:**

The binge alcohol intake was decreased in NLRP3 KO mice compared to the control mice. During alcohol withdrawal, NLRP3 deficiency attenuated anxiety-like behavior and neuronal injury in the mPFC and striatum. Moreover, we discovered that glutamatergic transmission to striatal neurons was reduced in NLRP3 KO mice. Importantly, in vivo optogenetic induction of long-term potentiation (LTP) of corticostriatal circuits reversed the effects of NLRP3 deficiency on glutamatergic transmission and anxiety behavior. We also demonstrated that optogenetic induction of LTD decreased anxiety-like behavior and caused a reduction in glutamatergic transmission. Interestingly, NLRP3 deficiency or inhibition (MCC950 injection) attenuated the anxiety-like behavior, but it did not prevent DID + gavage paradigm-induced a persistent enhancement of drinking in a two-bottle choice at 2 and 4 days into withdrawal.

**Conclusion:**

Our results demonstrate that NLRP3 deficiency decreases binge alcohol intake and anxiety-like behavior through downregulation of glutamatergic transmission in corticostriatal circuits, which may provide an anti-inflammatory target for treating alcohol use disorders.

**Supplementary Information:**

The online version contains supplementary material available at 10.1186/s12974-022-02666-w.

## Introduction

Alcohol use disorders (AUDs) are complex brain diseases caused by repeated excessive levels of drinking and increased vulnerability to relapse [1, 2]. Excessive chronic alcohol exposure also induces neuroninflammatory responses and anxiety upon alcohol cessation [3, 4]. Although the inflammatory response is involved in AUDs [5], the exact direct evidence of neuroinflammation for alcohol drinking and withdrawal-induced anxiety behavior remains unclear. Therefore, exploring neuroinflammatory function in the progression of alcohol drinking and addictive-related behavior is crucial for understanding the formation of AUDs.

Changes in neuroimmune-related gene expression after chronic alcohol consumption have been identified in murine and human brains [6]. This indicates that neuroinflammation is inextricably linked to AUDs [7]. The NOD-like receptor family pyrin domain containing-3 (NLRP3) inflammasome is a key molecular complex that orchestrates innate immune responses [8]. Notably, activation of the inflammasome pathway, particularly the NLRP3 inflammasome, plays a vital role in the regulation of the alcohol-induced neuroimmune response [9]. Activation of the inflammasome results in the activation of caspase-1 and an increase in proinflammatory cytokines, e.g., maturation of pro-IL-1β to IL-1β [10]. During alcohol exposure, proinflammatory cytokine production is associated with the activation of NLRP3 inflammasome proteins [11]. For example, the expression of the NLPR3 inflammasome and IL-1β is upregulated in the hippocampal area during alcohol exposure [12]. In mice genetically deleted with NLRP3, alcohol administration failed to induce the activation of caspase-1 and IL-1β [13]. These findings suggest that the NLRP3-IL-1β signaling cascade plays an important role in alcohol-induced neuroinflammation. In addition, neuroinflammation underlies the mechanisms of alcohol-induced neurotoxicity. Animal studies have shown that neurotoxicity is accompanied by impaired brain function, which induces withdrawal behaviors [14], such as anxiety, upon the cessation of alcohol consumption [15]. Indeed, excessive alcohol drinking elicits withdrawal anxiety behavior [16], which is a source of negative reinforcement for alcohol abuse. To determine whether the inflammasome plays a key role in this process, it is essential to target the inflammasome during alcohol drinking and withdrawal behavior.

An increasing numbers of studies have shown that the cortex, i.e., the medial prefrontal cortex (mPFC), and striatum are involved in alcohol-induced seeking behaviors [17, 18]. Optogenetic induction of long-term potentiation (LTP) in this circuit increases alcohol intake, while long-term depression (LTD) decreases alcohol intake [19], suggesting that the change in glutamatergic transmission in corticostriatal circuits is responsible for alcohol drinking and related behaviors. AMPA and NMDA receptors are the major ionotropic glutamate receptors in the brain. The NMDA receptor (NMDAR) is a well-characterized target of alcohol [20]. Chronic alcohol exposure leads to an upregulation of NMDAR function, which plays a hyperexcitable role and alters anxiety-like behavior [21]. The NMDAR-mediated responses change in corticostriatal circuits, which may be associated with alcohol withdrawal anxiety behavior [22]. Given that the function of glutamatergic transmission in corticostriatal circuits change with anxiety behavior, together with inflammasome cascade signaling in alcohol exposure, we sought to clarify the effects of the NLPR3 inflammasome on glutamatergic transmission in neuronal circuits and behavior.

In the present study, we aimed to define the role of the NLRP3 inflammasome in regulating alcohol intake and anxiety-like behavior. We discovered that binge alcohol consumption contributed to alcohol withdrawal anxiety-like behavior. NLRP3 deficiency decreased binge alcohol intake and binge drinking-induced anxiety-like behavior. We discovered the potentiated glutamatergic transmission was reduced in NLRP3 KO mice. Importantly, in vivo optogenetic induction of LTP or LTD in corticostriatal circuits (mainly mPFC-striatum) revealed that NLRP3 deficiency drove glutamatergic transmission, thereby affecting withdrawal anxiety-like behavior. Interestingly, while NLRP3 deficiency and pharmacological inhibition of NLRP3 reduced the expression of inflammatory factors, but it did not change DID + gavage paradigm-caused persistent increasing of drinking after withdrawal. Taken together, these findings demonstrate that NLRP3 inflammation affects alcohol intake and anxiety-like behaviors, providing an anti-inflammatory target to treat alcohol use disorders.

## Methods

### Animals

C57BL/6 (wild type) mice (8 weeks old) purchased from the Animal Core Facility of Nanjing Medical University were used for behavioral test. NLRP3 KO mice were a gift from Qiulun Lv’s lab at Nanjing Medical University (Nanjing, China). Animals were housed individually, under a 12-h light:dark cycle, with lights on at 7:00 a.m. Food and water were provided ad libitum. Mouse body weight (BW) was recorded weekly. All animal care and experimental procedures were approved by Nanjing Medical University Institutional Animal Care and Use Committee (IACUC-2103028) and were conducted in accordance with the Chinese National Institute of Guiding Principles for the Care and Use of Laboratory Animals.

### Reagents

MCC950 was purchased from Selleck (Selleck, USA). Polyclonal rabbit Caspase-1 (1:1000 dilution; 22915-1-AP, Proteintech), polyclonal mouse NLRP3 (1:1000 dilution; AG-20B-0014, Adipogen), monoclonal mouse GAPDH (1:10,000 dilution; 60004-1-lg, Proteintech). The blots were developed with horseradish peroxidase-conjugated secondary antibodies (anti-mouse IgG, 1:10,000 dilution, 7076S, CST; anti-rabbit IgG, 1:10,000 dilution, 7074S, CST). rAAV-hSyn-ChrimsonR-tdTomato-WPRE-hGH pA (4.93 × 10^12^ vg/mL) and rAAV-hSyn-tdTomato-WPRE-hGH pA (5.55 × 10^12^ vg/mL) were purchased from BrainVTA (Shumi Company, China). Nissl staining kits were purchased from Solarbio (Solarbio, China). ELISA kits were purchased from Meimian (Meimian, China). The other reagents were obtained from Sigma.

### Behavioral procedures

#### Drinking in the dark

Mice were weighed on the first day of each week that a new fluid was offered. In session 1, on Days 1, 2, and 3, the water bottle was replaced with a tube containing 20% alcohol in tap water (v/v) and volume was recorded in the dark cycle. 2 h later, the volume consumed was recorded and the alcohol tube was replaced with the water bottle. On Day 4, mice were offered alcohol for 4 h. Mice then had free access to water for the next 3 days. In session 2 and session 3, the same procedure was followed.

#### 2 h/4 h binge drinking

2 h/4 h binge drinking was performed in dark cycle. The water bottle was replaced with a tube containing 20% alcohol in tap water (v/v). The volume consumed was recorded for 2 h and 4 h drinking during the dark cycle.

#### Two-bottle choice for 2 h/4 h/24 h voluntary drinking

Eight-week-old male mice were single-housed under a 12:12 h light-dark cycle. Mice were given 2 h, 4 h, and 24 h voluntary drinking at 2 and 4 days into withdrawal. 24 h drinking was determined similarly as a two-bottle choice drinking paradigm. On the test day, mice were singly housed and given access to one bottle of 20% v/v ethanol and one bottle of water. The bottles were weighed before and after the test, respectively, to record the water and alcohol intake. The mice were weighed at the beginning. The position (left or right) of each solution was alternated as a control for side preference. 2 h/4–h voluntary drinking was performed in light cycle.

#### Gavage

Mice were administered 25% alcohol for 3 days directly to the stomach via oral gavage at a dose of 5 g/kg after DID training. Control mice were treated with water.

#### Open field test

The open field test was used to evaluate the level of anxiety in binge drinking model mice. The open field consisted of a box (40 × 40 × 40 cm); the mice were placed in the middle of the box at the beginning of the test. Then, their behavior was recorded on video for 15 min. The box was cleaned with 75% alcohol and dried between each experiment to remove odor. Recording and analysis of behavior was performed using Trackermaster software (Zongshi, Beijing, China).

#### Elevated plus maze

The elevated plus maze (EPM) comprised two open arms (30 × 5 cm), and two closed arms (30 × 5 × 25 cm) extending from the intersection zone (5 × 5 cm) and elevated to a height of 50 cm. Animals were transported to and habituated for 3 h in a preparation room. Animals were individually transported to the plus maze and placed on the center of the platform facing an open arm then allowed to freely explore the maze for 5 min. The plus maze was thoroughly cleaned with 75% alcohol and dried between each experiment to remove odor. Recording and analysis of behavior were performed using Trackermaster software (Zongshi, Beijing, China). Parameters scored manually from video recordings included: number of closed arm entries, number of open-arm entries, time spent on the open arm, time spent on the closed arm and velocity. For these parameters, an arm entry was recorded when all 4 paws had entered a single arm. Open arm entries expressed as a percentage of total entries, and the time spent on the open arms expressed as a percentage of total time spent on either the open or closed arms were used as measures of anxiety.

### Nissl staining

Following the behavior test, brain tissue was removed after killing and preserved in 4% formalin overnight before paraffin embedding. The striatum and the mPFC serial sections from each group were cut, mounted on plexiglass, and single-immunostained using cresyl fast violet for the histochemical demonstration of Nissl substances. For analysis, the minimal pixel was set at 50 pixels, and the average optical density values of Nissl bodies were analyzed. To calculate the relative optical density, the optical density of the other groups was divided by that of the WT group.

### Enzyme-linked immunosorbent assay

First, we extracted the mPFC and striatum from the mice brain tissue after the behavior test (Fig. 3) and after the last alcohol exposure (Fig. 6). Then, the mPFC and striatum were centrifuged at 12,000 r/min for 15 min and the clear supernatant was collected. Levels of IL-1β and TNF-α were detected using commercially available ELISA kits.

### Western blotting

The brains were removed, and the mPFC and striatum were carefully dissected. The proteins were separated by SDS-polyacrylamide gel electrophoresis, probed with antibodies and visualized by an enhanced chemiluminescence substrate system (Tanon, China). The protein bands were quantitatively analyzed by Image J.

### Stereotaxic surgery

Stereotaxic viral infusions were performed as follows. Mice were anesthetized using isoflurane and mounted in a rodent stereotaxic frame. The skin was opened to uncover the skull and expose Bregma and Lambda, and the location of the desired injection site. Small drill holes were made in the skull at the appropriate coordinates, according to the Paxinos atlas [23]. A microinjector was loaded with 0.4 µL of virus, and then lowered into the mPFC (AP: 1.94 mm, ML: ± 0.40 mm, DV: − 2.20 mm). This virus was infused into the brain at a rate of 0.08 µL/min. To avoid backflow of the virus, microinjectors were left in place for 10 min after the infusion was complete. After virus injections, bilateral optical fiber implants (300-μm core fiber secured to a 1.25-cm ceramic ferrule with 3 mm of fiber extending past the end of the ferrule) were lowered into the striatum right on the top of virus injection sites. Coordinates: AP, 1.00 mm; ML, ± 1.80 mm; and DV, − 3.00 mm. Implants were secured on the skull using metal screws and dental cement (Henry Schein) and covered with denture acrylic (Lang Dental). The incision was closed around the head cap and the skin Vet-bonded to the head cap. Mice were monitored for 1 week or until they resumed normal activity.

### In vivo LTP and LTD induction and in vitro optogenetic electrophysiology

One week after stereotaxic viral injection, mice were treated with 3 weeks of DID and 3 days of gavage alochol drinking. In vivo optogenetic stimulation were performed after 24 h withdrawal from DID + gavage model. An LTP/LTD-inducing protocol was delivered by optogenetic stimulation system (Thinker Tech Nanjing Biotech, China) 30 min before the open field test. LTP induction using the following protocol: 100 pulses at 50 Hz of 590-nm light (2 ms, 3 mW), repeated four times with 18-s intervals. The protocol was repeated three times with 5-min intervals. LTD induction employed the following protocol: 900 pulses at 1 Hz of 590-nm light (2 ms, 3 mW).

After behaviral tests, mice were killed and the brain slices from the striatum were prepared for in vitro optogenetic electrophysiology. For optogenetic stimualtion, light stimulation (Thorlabs at 625 nm, 2 mW) through the objective lens evoked glutamatergic transmission from cortex to striatum (mPFC-striatum). The paired-pulse ratios (PPRs) of AMPAR-mediated excitatory postsynaptic currents (EPSCs) were obtained using optogenetic stimulation at an interval of 100 ms. For measurement of the NMDAR/AMPAR ratio, the peak currents of AMPAR-mediated EPSCs were measured at a holding potential of − 70 mV and the NMDAR-mediated EPSCs were estimated as the EPSCs at + 40 mV, 50 ms after the peak AMPAR-EPSCs. The NMDA/AMPA ratio was calculated by dividing the NMDAR-EPSC by AMPAR-EPSC. The input–output relationships for AMPAR-EPSCs were measured at 5 different stimulating intensities. All the experiments were conducted in the presence of the GABA_A_ receptor antagonist, bicuculline (10 µM).

### Histology

The histology procedure was performed as follows. Mice were anesthetized and perfused intracardially with 4% paraformaldehyde in phosphate-buffered saline (PBS). Whole brains were taken out and put into 4% paraformaldehyde in PBS for post-fixation overnight (4 °C), then placed to 30% sucrose in PBS (4 °C) and allowed to sink to the bottom of the container before preparing for sectioning. Frozen brains were cut into 30-μm coronal sections on a cryostat. A fluorescence microscope (Nikon, Japan) was used to image these sections with a 590-nm laser (to excite tdT). All images were processed using Image J.

### Electrophysiology

Slice electrophysiology was performed as previously described [19, 24, 25]. Animals were anesthetized with isoflurane and killed after their last alcohol (or control water) consumption. 250-µm coronal sections containing the striatum were prepared in an ice-cold cutting solution containing (in mM): 40 NaCl, 148.5 sucrose, 4 KCl, 1.25 NaH_2_PO_4_, 25 NaHCO_3_, 0.5 CaCl_2_, 7 MgCl_2_, 10 glucose, 1 sodium ascorbate, 3 sodium pyruvate, and 3 myoinositol, saturated with 95% O_2_ and 5% CO_2_. Slices were then incubated in a 1:1 mixture of cutting solution and external solution at 32 °C for 45 min. The external solution contained the following (in mM): 125 NaCl, 4.5 KCl, 2.5 CaCl_2_, 1.3 MgCl_2_, 1.25 NaH_2_PO_4_, 25 NaHCO_3_, 15 sucrose, and 15 glucose, saturated with 95% O_2_ and 5% CO_2_. Slices were then maintained in an external solution at room temperature until use.

Slices were perfused with the external solution at a flow rate of 3–4 mL/min at 32 °C. The striatal neurons, mainly the DMSs, were identified and patched. The data were recorded using an IPA-2 integrated patch amplifier controlled with SutterPatch software (Sutter Instrument, Novato, CA, USA). For whole-cell voltage-clamp recordings, we used a Cs-based solution, containing (in mM): 119 CsMeSO_4_, 8 TEA.Cl, 15 HEPES, 0.6 EGTA, 0.3 Na_3_GTP, 4 MgATP, 5 QX-314.Cl, 7 phosphocreatine. The pH was adjusted to 7.3 with CsOH, with an osmolarity of 270–280 mOsm.

For electrical stimulation, bipolar stimulating electrodes were positioned 100–150 μm away from the recording neurons to elicit glutamatergic transmission in striatal neurons. The PPRs of AMPAR-mediated EPSCs were obtained using two electrical stimuli at an interval of 50 ms. All the experiments were conducted in the presence of the GABA_A_ receptor antagonist, bicuculline (10 µM).

### MCC950 treatment

MCC950, an NLRP3 inflammasome inhibitor, at 10 mg/kg was given to WT + alcohol mice on days 19–21, 0.9% saline was used for other groups through intraperitoneal injection.

### Statistical analysis

All data are expressed as the mean ± SEM. Statistical significance was assessed using the unpaired or paired *t* test, one-way ANOVA, and two-way RM ANOVA followed by Student–Newman–Keuls (SNK). Statistical significance was set at *p* < 0.05.

## Results

### Reduction of binge drinking intake in NLRP3 knockout mice

**Fig. 1:**
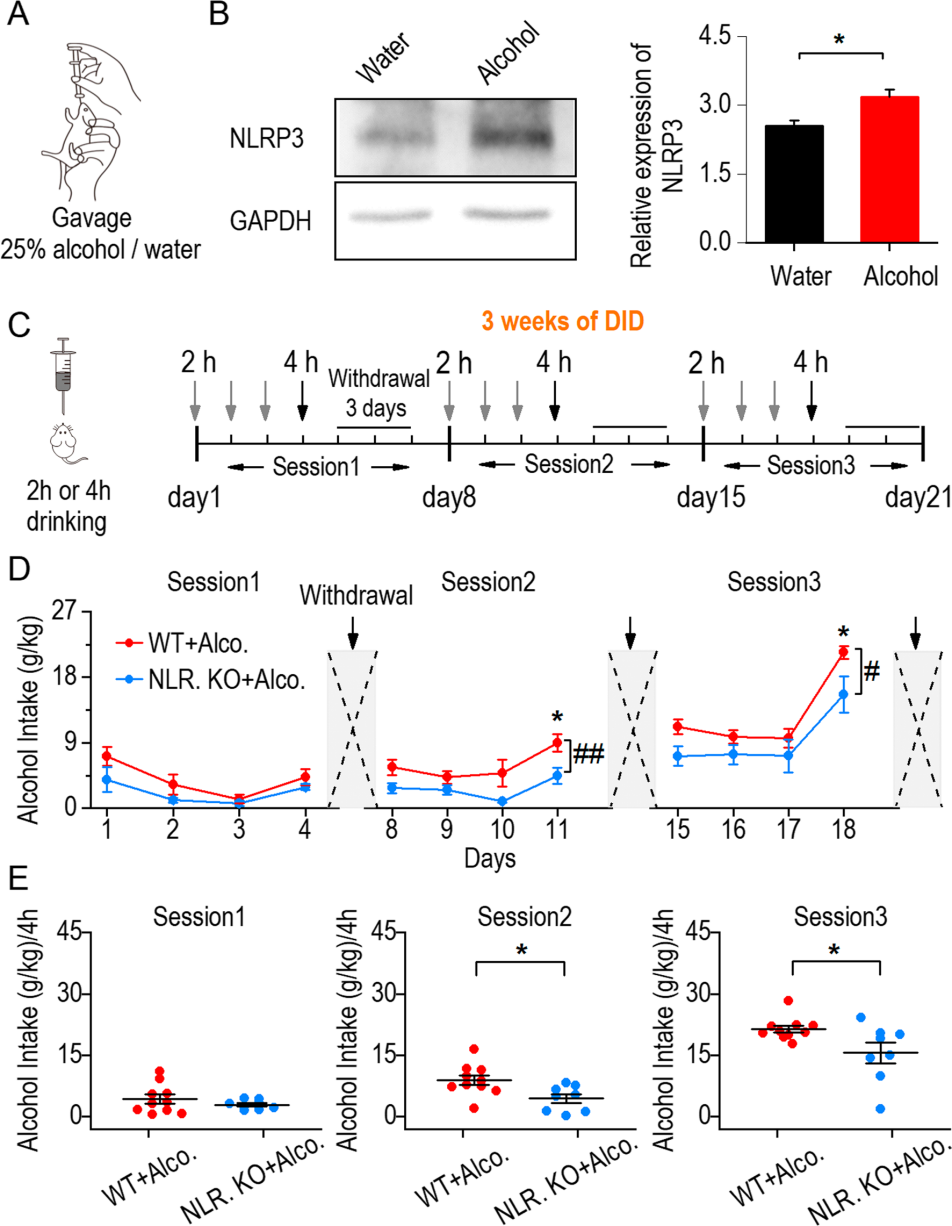
Reduction of binge drinking intake in NLRP3 knockout mice. **A** Schematic diagram depicting the gastric perfusion. C57BL/6 mice received either 25% alcohol or water via gavage. **B** Left, the samples showing the expression of the NLRP3 in water and alcohol group. Right, the relative expression of NLRP3 increased in mice gavaged with alcohol, *p < 0.05, unpaired *t* test, *n* = 3 mice (Water) and 3 mice (Alcohol). **C** Schematic diagram of the DID procedure. Left, mice were limited to acquire 20% alcohol through a needle tubing for 2 h or 4 h during three training sessions. Right, 3 weeks of DID procedure. For each training session, mice were given 2 h of alcohol consumption on the 1st–3rd day, 4 h of alcohol consumption on the 4th day, and abstinence treatment on the 5th–7th day. **D** Alcohol intake was lower in NLRP3 KO + alcohol group than WT + alcohol group at 4 h in session 2 and session 3. ^#^*p* < 0.05, ^##^*p* < 0.01, two-way RM ANOVA, *n* = 10 mice (WT + Alcohol) and 8 mice (NLRP3 KO + Alcohol). **E** The summary of 4-h alcohol intake in both WT + alcohol and NLRP3 KO + alcohol group from session 1 to 3. *p < 0.05, SNK in two-way RM ANOVA. Data are presented as mean ± SEM

Previous studies have shown that chronic alcohol exposure can activate the NLRP3 inflammasome [26]. To determine whether binge drinking induces changes in the NLRP3 inflammasome, wild-type mice were divided into two groups and given either water or 25% alcohol via gavage (Fig. 1A). Western blotting showed the expression of NLRP3 6 h after mice were gavaged with alcohol or water (Fig. 1B left and Additional file 1: Fig. S1). We found that the expression of NLRP3 was higher in the alcohol group than in the water group (Fig. 1B right). To investigate the effects of NLRP3 on alcohol intake, NLRP3 KO mice were used (Additional file 1: Fig. S2) in the drinking-in-the-dark (DID) procedure. For DID training, wild-type mice and NLRP3 KO mice were both trained with scheduled 2 h and 4 h of drinking and 3 days of abstinence for 3 weeks (Fig. 1C). We defined the wild-type mice to WT + alcohol group and the NLRP3 KO mice to NLRP3 KO + alcohol group. The data showed that alcohol intake was gradually decreased in NLRP3 KO + alcohol compared to the WT + alcohol group, especially at 4 h of drinking in the last two sessions (Fig. 1D, E). The results demonstrate that the activation of NLRP3 inflammasome is associated with excessive alcohol consumption.

### NLRP3 deficiency decreased binge drinking-induced anxiety-like behavior

**Fig. 2:**
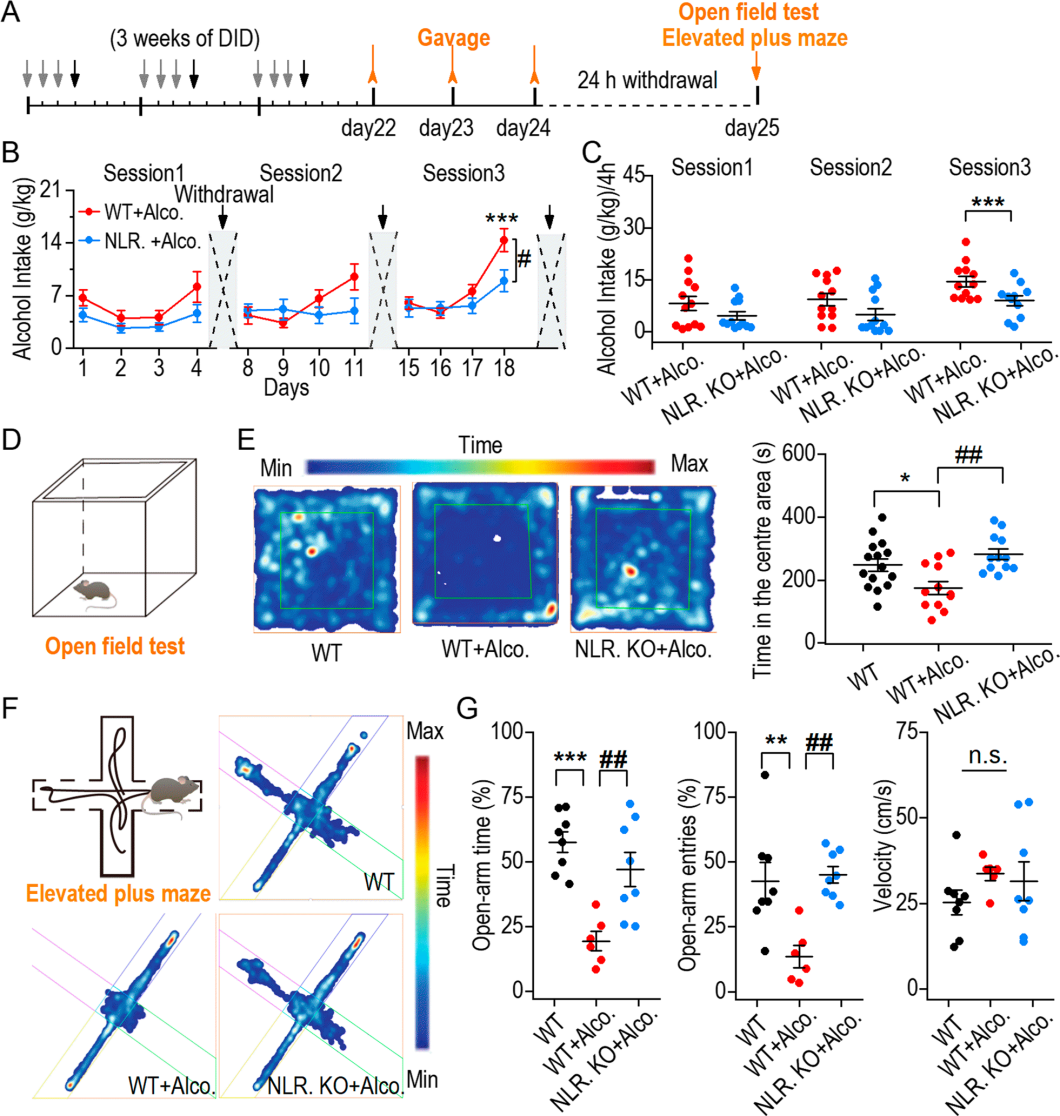
NLRP3 deficiency decreased binge drinking-induced anxiety-like behavior. **A** Schematic of the training procedure showing the mixed DID and alcohol gavage paradigm. **B** Alcohol consumption in WT + alcohol and NLRP3 KO + alcohol group during three training sessions. NLRP3 KO + alcohol group showed significantly lower alcohol drinking levels than WT + alcohol group at 4 h in session 3. ^#^*p* < 0.05, two-way RM ANOVA, *n* = 12 mice per group. **C** The statistics of 4-h alcohol intake in each session. ***p < 0.001, SNK in two-way RM ANOVA. **D** Schematic diagram of open field test. **E** Left, the representative traces in WT, WT + alcohol and NLRP3 KO + alcohol group. Right, WT + alcohol group spent less time in the center area compared to WT group, while NLRP3 KO + alcohol group spent more time in the center area compared to WT + alcohol group. *p < 0.05 vs WT group, ^##^*p* < 0.01 vs WT + alcohol group, one-way ANOVA. **F** Schematic diagram of EPM and representative samples traces of each group in the EPM. **G** Summary of open-arm time (left), open-arm entries (middle) and velocity (right). **p < 0.01, ***p < 0.001 vs WT group, ^##^*p* < 0.01 vs WT + alcohol group, one-way ANOVA, *n* = 8 mice (WT), 6 mice (WT + Alcohol) and 8 mice (NLRP3 KO + Alcohol). Data are presented as mean ± SEM

It has been widely verified that frequent bouts of intoxication/withdrawal of binge drinking causes anxiety [27, 28]; however, whether NLRP3 is involved in binge drinking-induced anxiety behavior remains unclear. By adding alcohol via gavage to DID, we aimed to investigate how the same amount of excessive alcohol exposure affected the behavior of mice in the WT + alcohol, NLRP3 KO + alcohol, and control groups. In this modified model, we treated WT + alcohol and NLRP3 KO + alcohol mice with 3 weeks of DID training, and then gavaged them with 25% alcohol for 3 days. Twenty-four hours after the last gavage, the open field test and EPM were used to detect anxiety-like behavior (Fig. 2A). Another cohort of mice different from the animals used in Fig. 1 were used to repeat this procedure. We found that alcohol intake in the NLRP3 KO + alcohol group was lower than that in the WT + alcohol group (Fig. 2B). There was a significant difference in alcohol intake at 4 h of drinking between the NLRP3 KO and WT + alcohol groups in the third session (Fig. 2C). After treatment with 25% alcohol by gavage for 3 days, anxiety-like behavior tests were conducted. In the open field test (Fig. 2D), mice in the WT + alcohol group spent less time in the center area than that in the WT group. NLRP3 KO + alcohol mice spent more time in the center area than WT + alcohol group mice, which indicated less anxiety after binge drinking (Fig. 2E). In the EPM test (Fig. 2F), NLRP3 KO mice spent more time in the open-arm and accessed the open-arm more than WT + alcohol group mice (Fig. 2G left and middle). The average velocity was not different among the groups (Fig. 2G right). In addition, there was no significant difference of the total distance in three group mice during open field and EPM tests (Additional file 1: Fig. S3A, B). To verify the genotype difference between wild-type and NLPR3 KO control mice, we tested the baseline behaviors in open field and EPM tests (Additional file 1: Fig. S3C, D). Taken together, these data suggest that NLRP3 deficiency decreases binge drinking-induced anxiety-like behavior.

### NLRP3 cascade signaling and neuronal injury in the mPFC and striatum occurred after binge drinking-induced anxiety-like behavior

**Fig. 3:**
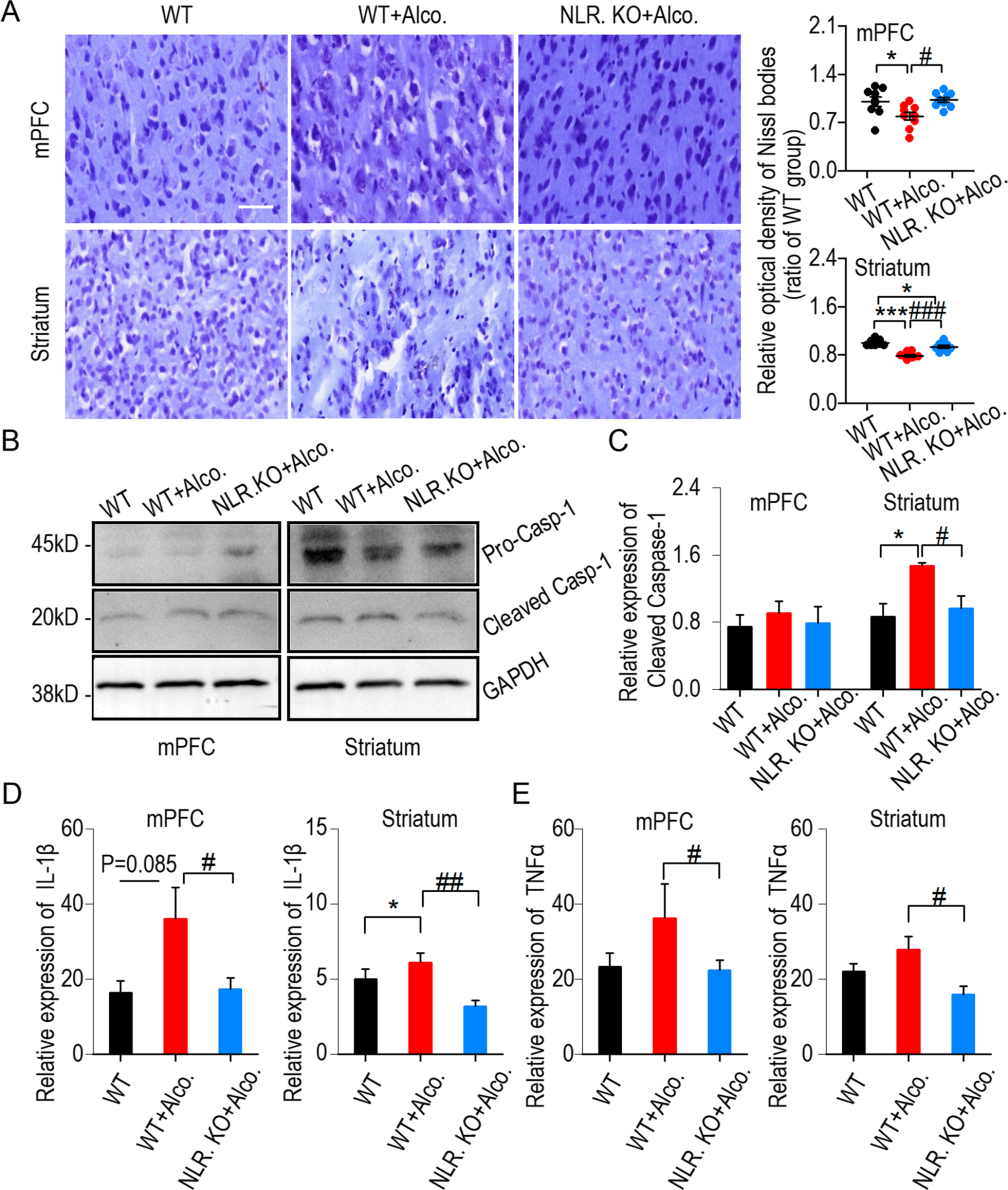
NLRP3 cascade signaling and neuron injury in the mPFC and striatum occurred after binge drinking-induced anxiety-like behavior. **A** Nissl bodies were stained by the Nissl staining. Scale bar = 50 μm. WT + alcohol group showed less Nissl bodies than the WT group and NLRP3 KO + alcohol group displayed more Nissl bodies than the WT + alcohol group in both mPFC and striatum, *p < 0.05, ***p < 0.001 vs WT group, ^#^*p* < 0.05, ^###^*p* < 0.001 vs WT + alcohol group, one-way ANOVA, 3 mice per group, 3 slices per mice. **B** The samples showing the expression of caspase-1 in the mPFC (left) and striatum (right). **C** The statistics of the expression of caspase-1 in the mPFC (left) and striatum (right), *p < 0.05 vs WT group, ^#^*p* < 0.05 vs WT + alcohol group, one-way ANOVA. **D** Histogram summary of expression of IL1-β in the mPFC and striatum; *p < 0.05 vs WT group, ^#^*p* < 0.05, ^##^*p* < 0.01 vs WT + alcohol group, one-way ANOVA, *n* = 3–7 mice for each group. **E**. Histogram summary of TNFα in the mPFC and striatum, ^#^*p* < 0.05 vs WT + alcohol group, one-way ANOVA, *n* = 4–5 mice for each group. Data are presented as mean ± SEM

Having shown that NLRP3 deficiency decreases binge drinking-induced anxiety-like behavior, we asked about the specific inflammatory response mechanism behind it. The cortex (mainly mPFC) modulates anxiety behavior [29], and the striatum has a close responsivity to reward [30]. WT group, WT + alcohol group and NLRP3 KO + alcohol group were trained by binge drinking and behavior procedures as shown in Fig. 2A. The slices and tissues from the mPFC and striatum were prepared to detect neuronal injury. Our results showed that Nissl bodies from the mPFC and striatum were decreased in the WT + alcohol group, and knocking out NLRP3 alleviated the damage to Nissl bodies caused by binge drinking (Fig. 3A). We next detected the expression of caspase-1 in the mPFC and striatum. The results showed that cleaved caspase-1 was activated after binge drinking in the striatum of WT + alcohol group, and was barely expressed in the NLRP3 KO + alcohol group (Fig. 3B, C and Additional file 1: Figs. S4, S5). We also extracted tissue supernatant to detect the relative expression of IL1-β and TNFα in the mPFC and striatum. ELISA showed that the relative expression of IL1-β in WT + alcohol group was an increasing trend (*P* = 0.085) than that in WT group in the mPFC (Fig. 3D left), and was higher than that in WT group in striatum (Fig. 3D right). Notably, there was a significant decrease of IL1-β in the NLRP3 KO + alcohol group compared to the WT + alcohol group in both the mPFC and striatum (Fig. 3D). Although there was no significant difference in TNFα between the WT group and the WT + alcohol group, a significant decrease of TNFa was found in the NLRP3 KO + alcohol group compared to the WT + alcohol group in both mPFC and striatum (Fig. 3E). In addition, using Nissl staining and western blotting, we also tested the genotype difference between wild-type and NLPR3 KO control mice (Additional file 1: Fig. S6). These results showed that NLRP3 plays an important role in inducing inflammatory cascade signaling and neuronal injury.

### NLRP3 deficits decreased the potentiated NMDAR-mediated glutamatergic transmission caused by binge drinking

**Fig. 4:**
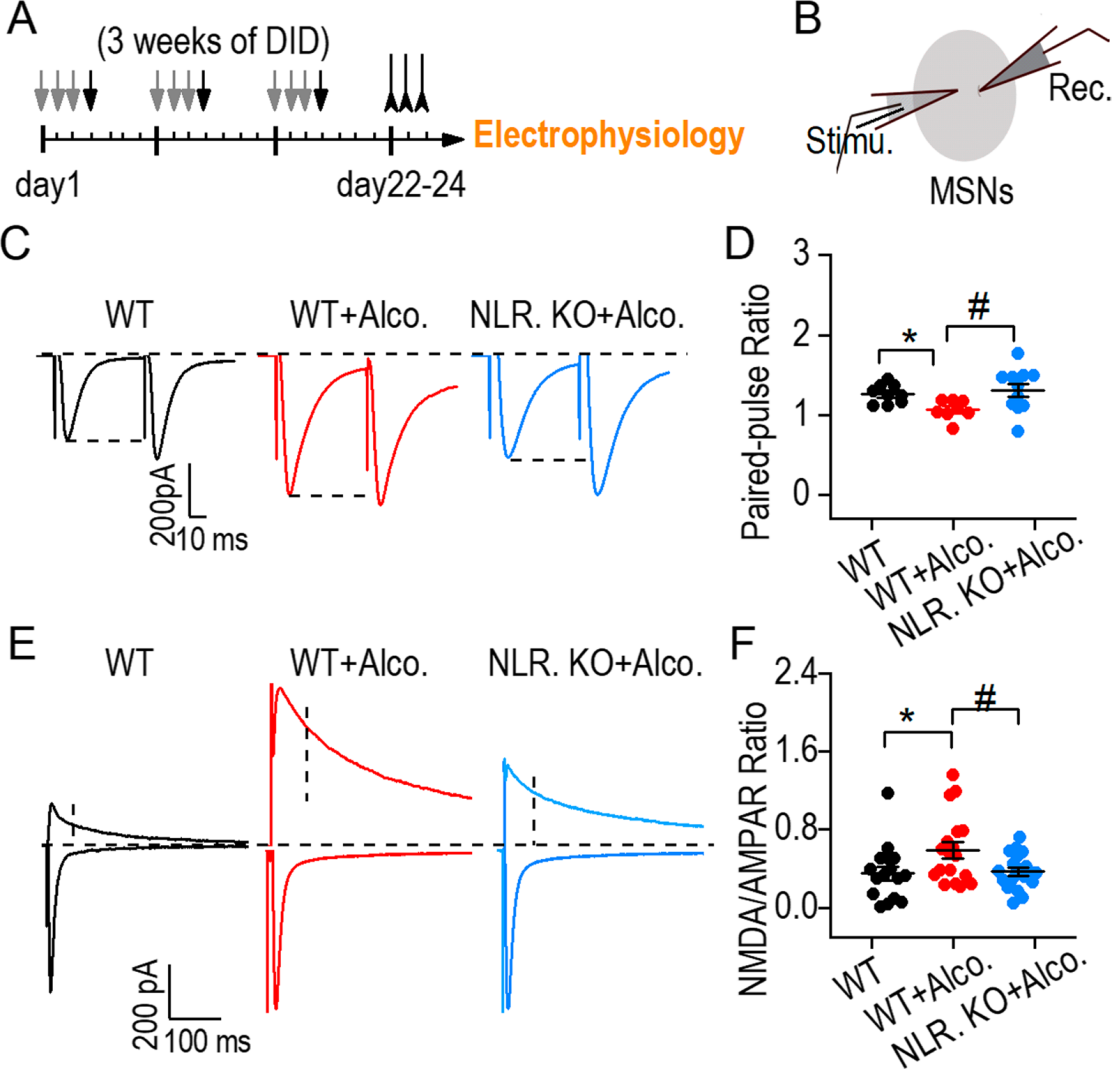
NLRP3 deficiency decreased the potentiated NMDAR-mediated glutamatergic transmission caused by binge drinking. **A** Experimental timeline of DID + gavage training, behavior and electrophysiology tests. **B** Schematic illustration of the electrical stimulation and the recording of striatal neurons. **C** Sample traces showing paired-pulse ratios from three groups. **D** Averaged data illustrating a decrease of PPR after binge drinking and an increase in NLRP3 KO + alcohol group compare to WT + alcohol group. *p < 0.05 vs. WT group, ^#^*p* < 0.05 vs WT + alcohol group, one-way ANOVA, *n* = 8–11 neurons. **E** Sample traces showing NMDAR/AMPAR ratio from three groups. **F** Averaged data showing the NMDAR/AMPAR ratio was significantly increased in WT + alcohol group compared to the WT group. The ratio was decreased by NLRP3 KO treatment compared to the WT + alcohol group. *p < 0.05 vs. WT group, ^#^*p* < 0.05 vs. WT + alcohol group, one-way ANOVA, *n* = 16–20 neurons. Data are presented as mean ± SEM

After observing the inflammatory response and neuronal injury in the striatum induced by NLRP3 after binge drinking-induced behaviors, we examined whether binge drinking could affect the glutamatergic transmission of striatal neurons. The striatum receives the main glutamatergic transmission from the cortex. After binge alcohol withdrawal-behavior (Fig. 4A), glutamatergic transmission was recorded from striatal medium spiny neurons (Fig. 4B). We found that the paired-pulse ratio of striatal neurons in WT + alcohol group decreased compared to the WT group, suggesting that the presynaptic mechanism of binge drinking induced excessive glutamatergic transmission. In contrast, the paired-pulse ratio of striatal neurons in the NLRP3 KO + alcohol group increased compared to that in the WT + alcohol group, indicating that NLRP3 greatly decreased the excessive glutamatergic transmission (Fig. 4C, D). We further measured the NMDAR/AMPAR ratio in striatal neurons from the three groups (Fig. 4E). We discovered that knocking down NLRP3 reduced the NMDAR/AMPAR ratio induced by binge drinking, suggesting that NLRP3 induced excessive glutamatergic transmission in striatal neurons (Fig. 4F). Together with genotype difference of glutamatergic transmission in WT and NLPR3 KO control mice (Additional file 1: Fig. S7), our data suggest that NLRP3 deficits decreased the potentiated NMDAR-mediated glutamatergic transmission caused by binge drinking.

### NLPR3 deficiency reduced anxiety-like behavior and modified the glutamatergic transmission to striatal neurons

**Fig. 5:**
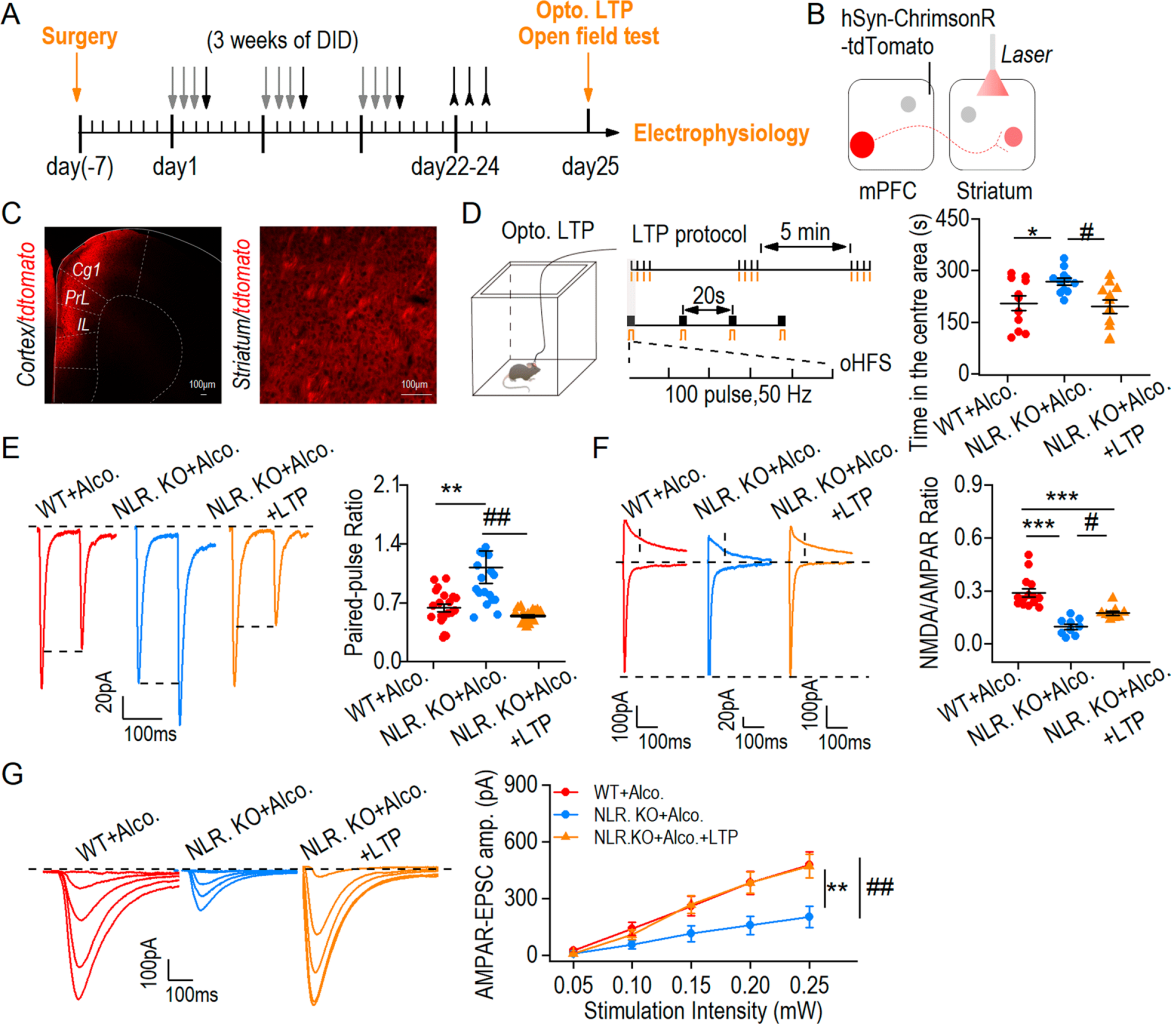
NLPR3 deficiency reduced anxiety-like behavior and modified the glutamatergic transmission to striatal neurons. **A** Experimental timeline showing the DID + gavage paradigm, optogenetic induction of LTP, behavior and electrophysiology procedures. **B** Schematic diagram of virus injection and optical fiber implantation. **C** Sample image of virus expression in the mPFC (left) and projection in the striatum (right). Scale bar = 100 μm. **D** Left, schematic diagram of open field test after LTP induction. Middle, high frequency stimulation protocol (100 pulses, 50 Hz). oHFS: optical high-frequency stimulation. Right, NLRP3 KO + alcohol mice with light-induced LTP spent less time in the center than NLRP3 KO + alcohol mice with no LTP. *p < 0.05 vs WT + alcohol group, ^#^*p* < 0.05 vs NLRP3 KO + alcohol group, one-way ANOVA, *n* = 12 mice (WT + Alcohol), 11 mice (NLRP3 KO + Alcohol) and 11 mice (NLRP3 KO + Alcohol + LTP). **E** Left, sample traces showing light stimulation of paired-pulse ratios (PPR) in striatal neurons. Right, analysis of the paired-pulse ratios detected in three groups. **p < 0.01 vs WT + alcohol group, ^##^*p* < 0.01 vs NLRP3 KO + alcohol group. **F** Left, sample traces showing light stimulation of NMDAR/AMPAR ratio in striatal neurons of three group mice. Right, analysis of the NMDAR/AMPAR ratios. The NMDAR/AMPAR ratio increased in NLRP3 KO with LTP induction compared to NLRP3 KO mice without optogenetic induction of LTP. ***p < 0.001 vs WT + alcohol group, ^#^*p* < 0.05 vs NLRP3 KO + alcohol group. **G** Left, sample traces showing light stimulation input–output curves (I/O) of AMPAR-EPSCs in three groups. Right, analysis of the amplitudes of AMPAR-EPSCs in each group. The AMPAR-EPSCs I/O was rescued by LTP compared to NLRP3 KO + alcohol group. **p < 0.01, ^##^*p* < 0.01. One-way ANOVA in **E**–**G**; *n* = 10–16 neurons from each group in **E**–**G**. Data are presented as mean ± SEM

We have shown that NLRP3 deficiency decreased glutamatergic transmission to the striatal neurons, and as the striatum primarily receives glutamatergic transmission from the cortex, we then asked whether LTP induction of corticostriatal circuits (mPFC-striatum) could prevent the effects of NLRP3 deficiency on anxiety behavior. The schematic diagram showed the behavior test, optogenetic stimulation and electrophysiological procedures (Fig. 5A). We first infused AAV-hSyn-ChrimsonR-tdTomato into the mPFC, and optical fibers were implanted into the striatum in mice (Fig. 5B, C). After recovery from surgery, the mice were divided into WT + alcohol, NLRP3 KO + alcohol, and NLRP3 KO + alcohol + LTP groups (LTP was applied to NLRP3 KO mice). Before the behavior test, an optogenetic protocol of high frequency stimulation (HFS) was induced in vivo to activate the cortex to striatum connectivity (Fig. 5D, left and middle). Compared to the WT + alcohol group, the NLRP3 KO + alcohol group spent more time in the center of the open field. Compared to the NLRP3 KO + alcohol group, the time spent in the center decreased in the NLRP3 KO + alcohol + LTP group of NLRP3 KO mice (Fig. 5D, right). This result suggests that light activation of the corticostriatal circuit pathway reversed NLRP3 deficiency-mediated anxiety-like behavior. To verify the above results, glutamatergic transmission from the cortex to the striatum was measured. Compared to the WT + alcohol group, optogenetic stimulation of the PPR increased in the NLRP3 KO + alcohol group. After inducing LTP, the PPR was significantly decreased in the NLRP3 KO + alcohol + LTP group (Fig. 5E), suggesting that HFS stimulation of corticostriatal circuits exerted a presynaptic mechanism on reversing glutamatergic transmission. We also found that compared to the NLRP3 + alcohol group, the NMDAR/AMPAR ratio after light stimulation was increased in the NLRP3 KO + alcohol + LTP group (Fig. 5F). Finally, we measured the input–output relationship of AMPA-mediated responses in the three groups. We observed that the AMPAR-EPSCs of NLRP3 KO + alcohol group was lower than that in the WT + alcohol group, and this effect was reversed after light-induced LTP activated the corticostriatal pathway (Fig. 5G). Then we asked whether the glutamatergic transmission is responsible for the behavior. The protocol of 900 pulses for optogenetic LTD was applied in corticostriatal circuits and we found that induction of LTD decreased glutamatergic transmission and anxiety-like behavior (Additional file 1: Fig. S8), indicating a link of the NLRP3 deficiency-induced reduction of alcohol withdrawal anxiety-like behavior. In addition, there was no significant difference of total distance in three groups (Additional file 1: Fig. S9A, B). To exclude the effects of opsin expression and potential laser heating, the control experiments were also tested (Additional file 1: Fig. S9C, D). Taken together, NLRP3 deficiency decreased glutamatergic transmission in corticostriatal circuits and decreased anxiety-like behavior.

### Alcohol withdrawal anxiety-like behavior accompanied by neuroinflammation further promoted binge drinking

**Fig. 6:**
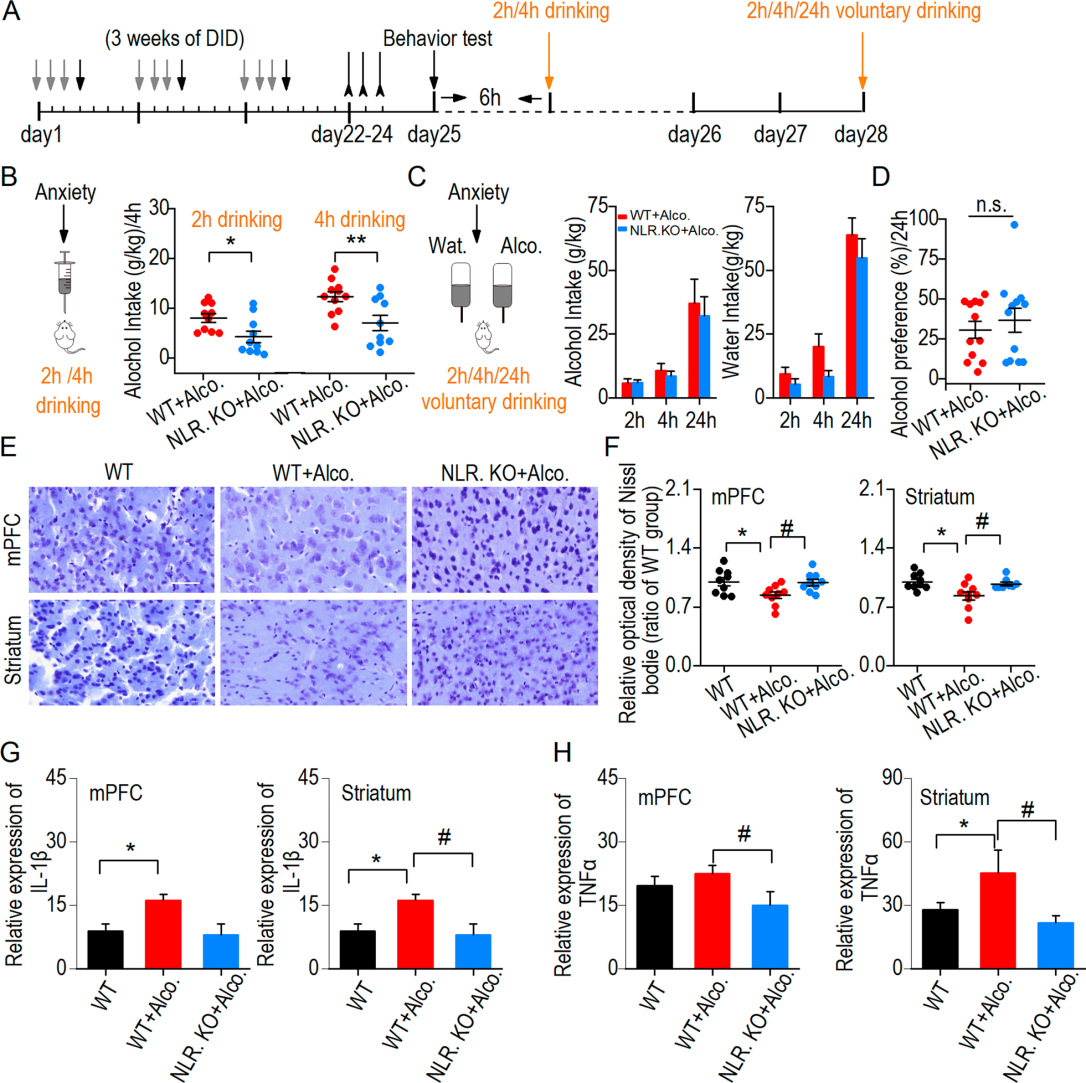
Alcohol withdrawal anxiety accompanied by neuroinflammation further promoted binge drinking. **A** Experimental timeline of DID + gavage training, behavior test and measurement of alcohol drinking. **B** Left, schematic diagram of 2 h/4 h binge drinking after anxiety-like behavior. Right, NLRP3 KO + alcohol group mice showed a decrease in alcohol intake at 2 h and 4 h compared to the WT + alcohol group. *p < 0.05, **p < 0.01 vs WT + alcohol group, unpaired *t* test. *n* = 12 mice (WT + alcohol) and 10 mice (NLRP3 KO + alcohol). **C** Left, schematic diagram of two-bottle choice at 2 h, 4 h and 24 h voluntary drinking at 4 days into withdrawal. Right, voluntary alcohol intake showed no difference between WT + alcohol and NLRP3 KO + alcohol group. *p* > 0.05, unpaired *t* test. **D** Alcohol preference showed no difference between WT + alcohol and NLRP3 KO + alcohol group at 24 h drinking. n.s. *p* > 0.05, unpaired *t* test. **E** Sample images showing Nissl bodies in three groups. Scale bar = 50 μm. **F** The NLRP3 KO + alcohol group displayed more Nissl bodies in the mPFC and striatum compared to the WT + alcohol group. ^#^*p* < 0.05 vs WT + alcohol group, one-way ANOVA; 3 mice per group. **G** The expression of IL1-β of the mPFC and striatum decreased in NLRP3 KO group compared the WT + alcohol group. *p < 0.05 vs WT group, ^#^*p* < 0.05 vs WT + alcohol group, one-way ANOVA. **H** NLRP3 deficiency inhibits the expression of TNFα in the striatum. *p < 0.05 vs WT group, ^#^*p* < 0.05 vs WT + alcohol group, one-way ANOVA. 3–6 mice per group in **G** and **H**. Data are presented as mean ± SEM

We wondered whether anxiety-like behavior could further change alcohol drinking. After anxiety-like behavior test, we measured 2 h/4 h binge drinking, and 24-h voluntary alcohol drinking at 4 days withdrawal (Fig. 6A). One day after withdrawal, the 2 h/4-h drinking alcohol intake in the NLRP3 KO + alcohol group was lower than that in the WT + alcohol group, suggesting that NLRP3 deficiency reduces alcohol consumption in binge drinking (Fig. 6B). Then, 4 days into withdrawal, a 2 h, 4 h, and 24-h voluntary alcohol drinking was recorded in the two groups mice (Fig. 6C, left). The results showed that neither alcohol intake nor water intake were different between the WT + alcohol and NLRP3 + alcohol groups (Fig. 6C, middle and right). There was also no difference in alcohol preference in 24 h voluntary alcohol drinking (Fig. 6D). These results suggest that alcohol withdrawal anxiety further promotes binge drinking and DID paradigm did not result in a persistent enhancement of drinking in the 2-bottle choice at 4 days into withdrawal.

We further identified neuronal injury and inflammatory responses after measuring alcohol intake. We found that compared to the WT group, Nissl bodies in both the mPFC and striatum were decreased in the WT + alcohol group. Nissl bodies in the NLRP3 KO + alcohol group were protected because of NLRP3 deficiency (Fig. 6E, F). ELISA was used to investigate the expression levels of IL1-β and TNFα in the mPFC and striatum. We found that the expression of IL1-β increased after chronic drinking in the mPFC and striatum. Comparing the NLRP3 KO + alcohol group to the WT + alcohol group, there was a significant decrease of IL1-β in the striatum, as explained by the knockout of the NLRP3 gene (Fig. 6G). The data also showed that the NLRP3 KO + alcohol group had lower expression of TNFα in the mPFC and striatum than the WT + alcohol group (Fig. 6H). All the results showed that NLRP3 deficiency could relieve the inflammatory response caused by chronic drinking. Together with the behavioral results, these data indicate that in our binge drinking paradigm, alcohol withdrawal anxiety accompanied by neuroinflammation further promotes binge drinking.

### Pharmacological inhibition of NLRP3 exerted a similar role to NLRP3 deficiency on anxiety-like behavior

**Fig. 7:**
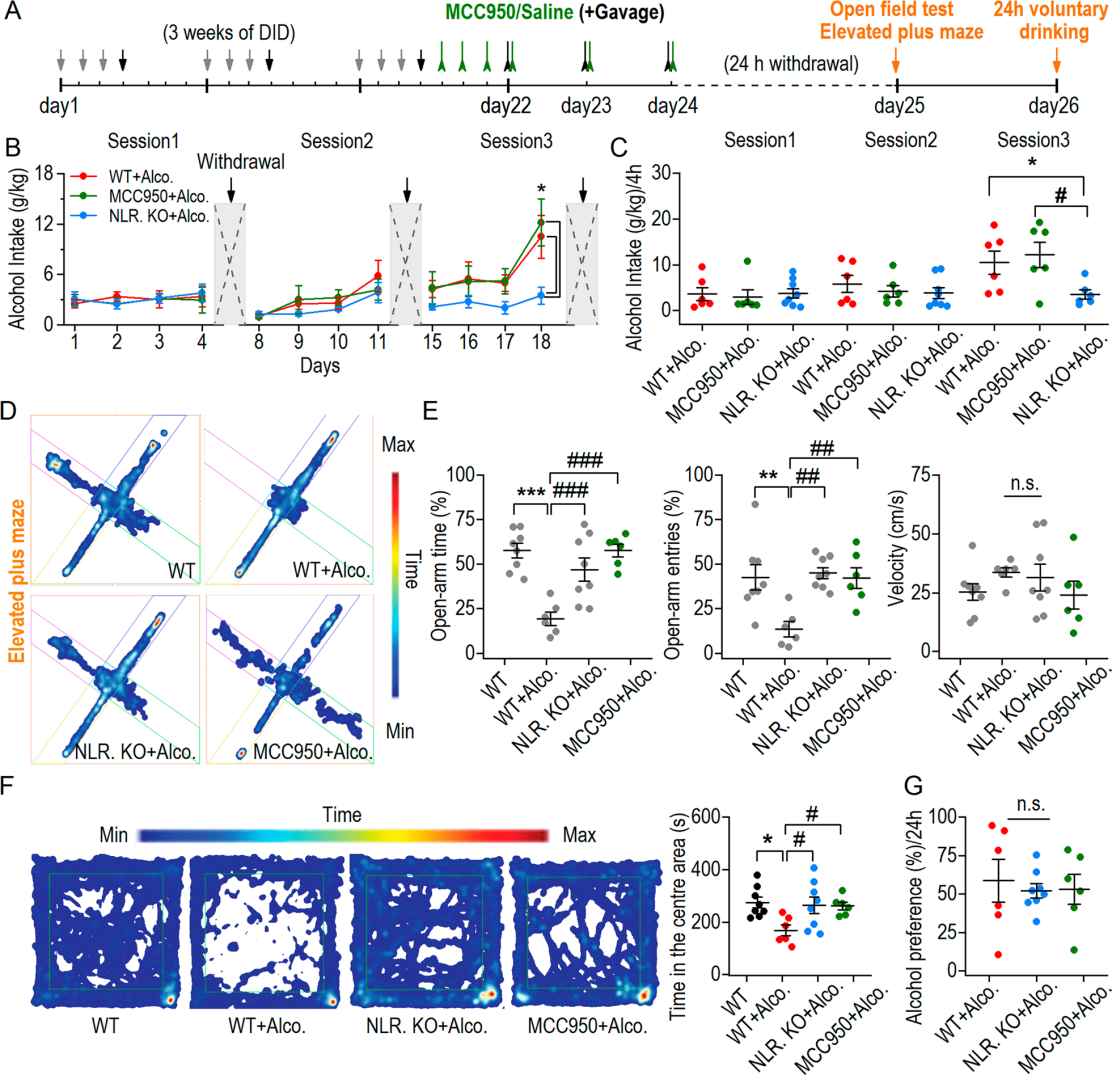
Pharmacological inhibition of NLRP3 exerts the similar role of NLRP3 deficiency on anxiety-like behavior. **A** Experimental timeline of DID training and alcohol gavage, NLRP3 inhibitor (MCC950) injection and behavior tests. **B** In 3 weeks of DID training, WT + alcohol group and MCC950 + alcohol group mice (untreated with MCC950) had higher alcohol consumption compared to the NLRP3 KO + alcohol group mice. *p < 0.05 vs WT + alcohol group, ^#^*p* < 0.05 vs MCC950 + alcohol group, two-way RM ANOVA; *n* = 6 mice (WT + Alcohol), 8 mice (NLRP3 KO + Alcohol) and 6 mice (MCC950 + Alcohol). **C** Statistics of 4-h alcohol intake in each session in DID model. *p < 0.05, ^#^*p* < 0.05, one-way ANOVA. **D** Samples traces of each group in the EPM. **E** Results of open-arm time, open-arm entries and velocity after DID + gavage drinking paradigm. **p < 0.01, ***p < 0.001 vs WT group, ^##^*p* < 0.01, ^##*#*^*p* < 0.001 vs WT + alcohol group, one-way ANOVA, *n* = 8 mice (WT), 6 mice (WT + Alcohol), 8 mice (NLRP3 KO + Alcohol) and 6 mice (MCC950 + Alcohol). WT group, WT + alcohol group and NLRP3 KO + alcohol group were referenced from Fig. 2G. **F** Samples traces of each group in the open field test and statistics of time spent in the center area. *p < 0.05 vs WT group, ^#^*p* < 0.05 vs WT + alcohol group, one-way ANOVA. **G** Alcohol preference of 24-h voluntary drinking showed no difference among the three groups at 2 days into withdrawal. n.s. *p* > 0.05, one-way ANOVA. Data are presented as mean ± SEM

To further identify the role of NLRP3 in drinking and behavior, an inhibitor of NLRP3, MCC950, was used. We tested the therapeutic effect of MCC950 on anxiety-like behavior and withdrawal-voluntary alcohol consumption (Fig. 7A). Alcohol intake in NLRP3 KO + alcohol group was lower than that in both the WT + alcohol and MCC950 + alcohol groups in DID model (Fig. 7B, C). Five days of MCC950 injection was performed in MCC950 + alcohol group mice at the end of DID training, we tested the effects of MCC950 on anxiety-like behavior and alcohol intake. We found that similar to NLRP3 KO + alcohol group, MCC950 + alcohol group mice spent more time in the open arm (Fig. 7D, E left) and accessed the open-arm more (Fig. 7D, E middle) than the WT + alcohol group in the EPM, with no difference in velocity between the groups (Fig. 7E right). In open field test, the MCC950 + alcohol group spent more time in the center area than the WT + alcohol group, which is similar to the NLRP3 KO + alcohol group (Fig. 7F). After testing anxiety-like behavior, we investigated the effects of MCC950 on 2 h/4 h/24 h voluntary drinking intake at 2 days of withdrawal. The data showed no difference in alcohol preference at 24 h of voluntary drinking in MCC950 + alcohol group, which is similar to that in NLRP3 KO + alcohol group (Fig. 7G). In summary, pharmacological inhibition of NLRP3 exerts a similar effect as NLPR3 deficiency on anxiety-like behavior. Both NLRP3 KO and MCC950 did not prevent a persistent enhancement of voluntary drinking at 2 days into withdrawal.

## Discussion

**Fig. 8:**
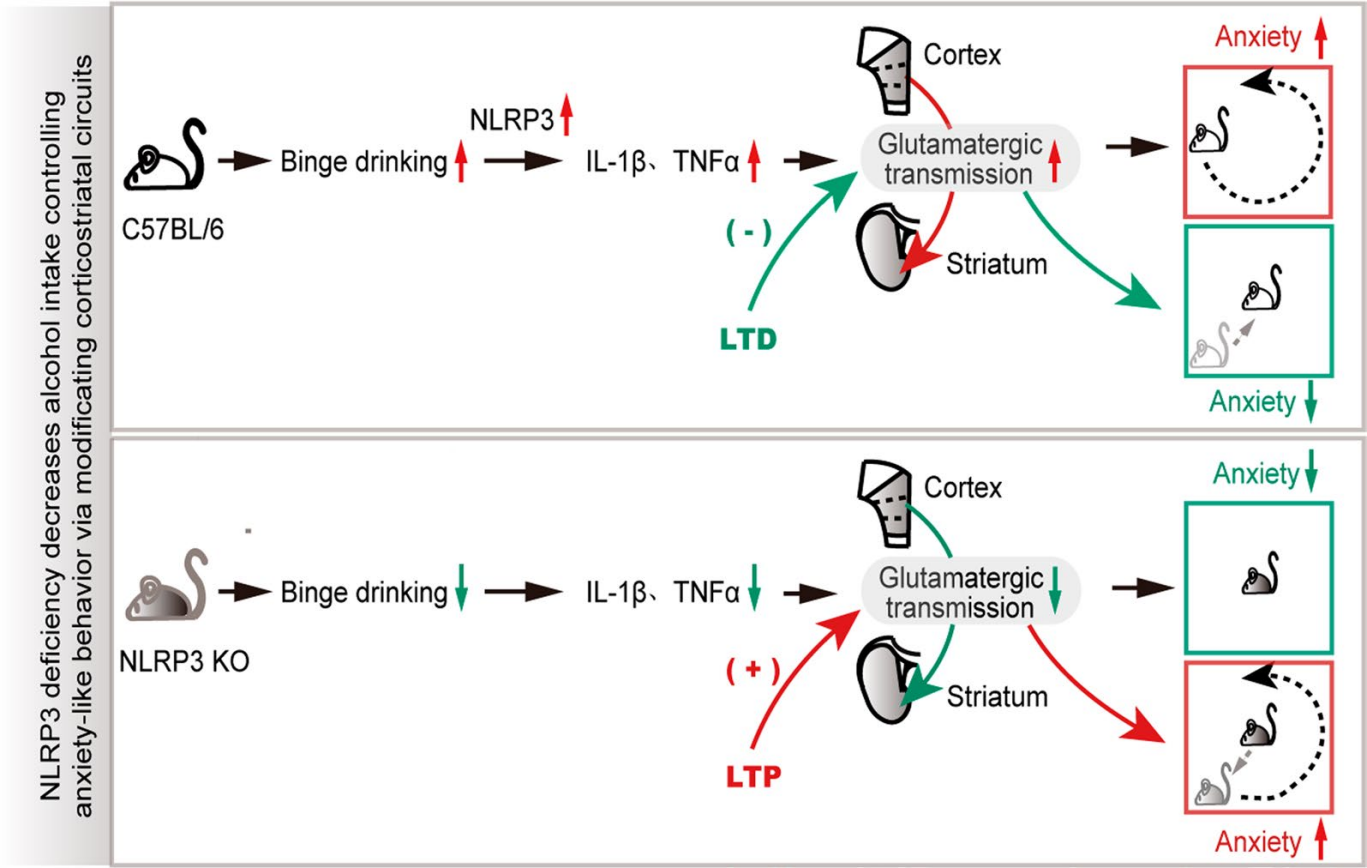
NLRP3 deficiency decreases alcohol intake controlling anxiety-like behavior via modification of glutamatergic transmission in corticostriatal circuits. Top, the NLRP3 inflammasome in C57BL/6 mice is activated via binge drinking, which also increases anxiety behaviors. Optogenetic induction of LTD in corticostriatal glutamatergic transmission prevents alcohol withdrawal anxiety-like behavior. Bottom, NLRP3 deficiency decreases its downstream inflammatory factor and alcohol intake, thereby preventing anxiety-like behavior. Optogenetic induction of LTP in corticostriatal circuits reversed the NLPR3 deficiency-reduction of glutamatergic transmission and anxiety-like behavior. It highlights the role of NLRP3 in binge drinking and anxiety-like behavior

In this study, we showed that binge alcohol treatment in mice resulted in an increase in alcohol intake and a withdrawal of anxiety-like behavior. Binge alcohol intake was mediated by the activation of the NLPR3 inflammasome in the brain area, and was decreased in NLRP3 KO mice. Importantly, the data of optogenetic LTP and LTD manipulation in glutamatergic transmissions demonstrated that NLRP3 deficiency contributed to the reversal of withdrawal symptoms via regulation of glutamatergic transmission in corticostriatal circuits. Our results highlight that NLRP3 deficiency decreased withdrawal-mediated binge alcohol intake but not voluntary drinking at 2 and 4 days into withdrawal. These data suggest that changes in glutamatergic transmission caused by the NLRP3 inflammasome in corticostriatal circuits modulate binge alcohol drinking-induced withdrawal behavior (Fig. 8), providing an anti-inflammatory strategy in binge drinking to treat alcohol use disorders.

Alcohol exposure alters the NLRP3 inflammasome, and neuroimmune signaling is involved in alcohol-seeking behavior. We found that binge alcohol drinking in the gavage model increased NLRP3 expression, which is different from a report that acute alcohol drinking-inhibited NLPR3 [31]. The reason might be the NLPR3 expression under the alcohol withdrawal conditions in the gavage model. We investigated the role of NLRP3 in alcohol intake using the well-established drinking-in-the-dark (DID) model [32]. The NLPR3 KO mice used in the DID model allowed us to study the effect of NLRP3 deficiency on binge drinking intake. Since adolescent binge drinking is sensitive to alcohol intake [33], we chose adult mice at 8 weeks in this study to avoid aging-preference alcohol intake. Interestingly, NLRP3 deficiency decreased alcohol intake with increasing drinking sessions in the DID model. This new finding suggests that the NLRP3 inflammasome is necessary for binge drinking. Since the DID model elicited withdrawal-anxiety behavior for a longer period [28], we modified this model to mix DID with the gavage model. The reason for using gavage is to exclude the effects of lower alcohol intake in NLRP3 KO mice on later behavioral tests. After the same amount of alcohol exposure in the gavage session, NLRP3 deficiency prevented withdrawal-induced anxiety-like behavior in the open field test and EPM. This result is consistent with reports that administration of alcohol failed to activate neuroinflammation that may induce anxiety-like behavior [13, 34].

The cortex (mainly mPFC) and striatum are implicated in alcohol consumption and anxiety behavior [35]. After binge and gavage drinking of alcohol-induced anxiety behavior, NLRP3 deficiency prevented neuronal injury in both the mPFC and striatum, which is consistent with reports that activation of inflammation results in neuronal damage [10, 13]. Alcohol triggers the innate immune response by activating NOD-like receptors, which causes the release of IL-1β and TNF-α, and the inflammasome cascade [9]. The crucial role of IL-1β and TNF-α in alcohol consumption is widely acknowledged. Breese’s research confirmed that IL-1β and TNF-α are involved in the sensitized anxiety response caused by repeated alcohol withdrawal [36]. The two studies also highlighted the vital role of IL-1β in modulating binge-like alcohol consumption and neuropathogenesis associated with alcohol dependence [9, 37]. Consistent with these findings, we found that NLRP3 deficiency decreased cleaved caspase-1 in the striatum, while it lowered this tendency in the mPFC. Accordingly, the expression of IL-1β and was TNF-α decreased in both the mPFC and striatum. These findings provide corroborating evidence that the NLRP3 inflammasome cascade contributes to alcohol injury. Many studies have demonstrated that chronic alcohol exposure changes glutamatergic transmission and drives alcohol-seeking behavior [19, 38, 39]. After anxiety-like behavior, NLRP3 deficiency reduced the presynaptic release of glutamate in the striatum, suggesting that NLRP3 affects glutamatergic transmission in the cortex and modulates anxiety-like behavior. NMDAR-mediated glutamatergic transmission is a target for alcohol [40]. Acute alcohol exposure inhibits NMDARs, while chronic alcohol exposure increases the activity of NMDAR responses. Binge alcohol withdrawal induced an increase in the NMDAR response, and NLRP3 deficiency prevented the activation of NMDARs, suggesting that NLRP3 signaling is associated with changes in glutamatergic transmission. Furthermore, the NLRP3 KO mice consumed less alcohol than the WT mice during the 3-week DID paradigm, which suggests that the difference in glutamatergic transmission may possibly be due to the NLRP3 KO mice’s lower alcohol consumption. It is consistent with reports suggesting long-term changes in NMDAR-mediated glutamatergic transmission were caused by chronic alcohol exposure [41–43]. Indeed, inflammation has been shown to regulate glutamatergic transmission in other diseases [44]. Therefore, NLRP3 depletion may decrease the inflammatory-mediated abnormal changes in glutamatergic transmission in neuronal circuitry.

Abbreviated glutamatergic transmission increases in the striatum following alcohol exposure. Optogenetic induction of LTP and LTD alters alcohol drinking behavior [19]; however, little is known about this synaptic plasticity in binge alcohol withdrawal anxiety behavior. Low-frequency stimulation at 1 Hz for LTD induction in the cortex to the striatum prevents anxiety-like behavior, suggesting that the enhancement of glutamatergic transmission drives alcohol withdrawal anxiety-like behavior. A report showed that LTD induction was associated with a transient increase in alcohol-seeking behavior [45]. The different conditions may be an explanation why our LTD protocol is applicable to alcohol withdrawal responses. HFS stimulation of corticostriatal circuits rescued the NLRP3 deficiency-induced reduction in anxiety-like behavior, providing a link between NLRP3 signaling and glutamatergic transmission. Although presynaptic HFS stimulation is not stable enough to induce LTP [46, 47], our results showed that this protocol caused an increase in glutamatergic transmission, suggesting that HFS stimulation is sufficient to drive potentiation, which is involved in inflammatory-mediated changes in glutamatergic transmission.

Numerous studies have revealed that an increase in anxiety is a common symptom of alcohol withdrawal and contributes to relapse [48]. The causes of withdrawal-induced binge drinking and compulsory drinking are still not clear. Thus, 2 h/4 h binge drinking and 2 h/4 h/24 h voluntary drinking were determined. Our findings demonstrated that NLRP3 deficiency decreased 2 h/4 h binge alcohol intake but not 2 h/4 h/24 h voluntary alcohol intake at 4 days into withdrawal. Although repeated excessive alcohol consumption induced persistent inflammatory responses and neuron injury, the withdrawal promoted binge drinking but did not result in a persistent enhancement of voluntary drinking 2 and 4 days into withdrawal. This could be attributed to the decline of anxiety-escalated drinking. Our findings showed that NLPR3 deficiency prevents binge alcohol intake and withdrawal anxiety-like behavior. This result is consistent with the concept that withdrawal behavior induces a relapse for alcohol [49]. In addition, pharmacological administration of the NLRP3 inhibitor MCC950 has the same effect as NLRP3 deficiency on anxiety-like behavior. Interestingly, previous research demonstrated that MCC950 reduces voluntary alcohol consumption in female but not in male mice [50], which is consistent with our report of MCC950 on voluntary drinking at 2 days into withdrawal. Our research provides a new effect of MCC950 in decreasing alcohol consumption in male mice through the DID paradigm. In addition, elucidating the mechanisms by which anxiety develops in female mice during binge drinking will be an interesting area of future research.

## Conclusions

In summary, we demonstrated that binge drinking intake changed in NLRP3 KO mice. NLRP3 deficiency reduced excessive drinking in mice exhibiting anxiety-like behaviors. Using optogenetic induction of LTP or LTD, our results showed the relationship between NLRP3 cascade signaling and glutamatergic transmission, providing evidence of NLRP3-mediated regulation in glutamatergic transmission, thereby affecting withdrawal anxiety-like behaviors. Our findings suggest that NLRP3 signaling is an essential regulator of AUDs and a therapeutic target for the treatment of alcohol withdrawal symptoms.

## Supplementary Information

**Additional file 1: Fig. S1.** The expression of NLRP3 and GAPDH. **Fig. S2.** Genotyping of NLRP3 KO mice. **Fig. S3.** Measurement of total distance and anxiety-like behavior in experimental and control groups. **Fig. S4.** The expression of caspase-1 and GAPDH in mPFC. **Fig. S5.** The expression of Caspase-1 and GAPDH in striatum. **Fig. S6.** The identification of genotype difference between wild-type and NLPR3 KO control mice in Nissl staining and Western blotting tests. **Fig. S7.** The difference of PPR and NMDAR/AMPAR ratio between WT and NLRP3 KO control group. **Fig. S8.** Optogenetic induction of LTD in corticostriatal glutamatergic transmission prevented alcohol withdrawal anxiety-like behavior. **Fig. S9.** Measurement of total distance and anxiety-like behavior of groups of mice as control in optogenetic LTP and LTD experiments.

## Data Availability

The datasets used and/or analyzed during the current study are available from the corresponding author on reasonable request.

## Abbreviations

Alco.: Alcohol
AMPAR: α-Amino-3-hydroxy-5-methyl-4-isoxazole-propionic acid receptor
AUDs: Alcohol use disorders
BW: Body weight
CFV: Cresyl fast violet
Ctrl.: Control
DID: Drinking in the dark
DMSs: Dorsomedial striatum neurons
ELISA: Enzyme-linked immunosorbent assay
EPM: Elevated plus maze
EPSCs: Excitatory postsynaptic currents
GABA: Gamma-aminobutyric acid
HFS: High-frequency stimulation
IL1-β: Interleukin-1β
LTD: Long-term depression
LTP: Long-term potentiation
NLRP3: NOD-like receptor family, pyrin domain containing 3
NLR. KO: NLRP3 gene knockout
NMDAR: *N*-Methyl-d-aspartic acid receptor
PBS: Phosphate-buffered saline
PPRs: Paired-pulse ratios
SDS: Sodium dodecyl sulfate
TNFα: Tumor necrosis factor alpha
SNK: Student–Newman–Keuls
