# Supplementary material for: NLRP3 deficiency decreases alcohol intake controlling anxiety-like behavior via modification of glutamatergic transmission in corticostriatal circuits
Source: J Neuroinflammation. 2022 Dec 20;19:308. doi: 10.1186/s12974-022-02666-w (PMC9764485; doi:10.1186/s12974-022-02666-w)
Supplement: Supplementary file 1 — Additional file 1: Fig. S1. The expression of NLRP3 and GAPDH. Fig. S2. Genotyping of NLRP3 KO mice. Fig. S3. Measurement of total distance and anxiety-like behavior in experimental and control groups. Fig. S4. The expression of caspase-1 and GAPDH in mPFC. Fig. S5. The expression of Caspase-1 and GAPDH in striatum. Fig. S6. The identification of genotype difference between wild-type and NLPR3 KO control mice in Nissl staining and Western blotting tests. Fig. S7. The difference of PPR and NMDAR/AMPAR ratio between WT and NLRP3 KO control group. Fig. S8. Optogenetic induction of LTD in corticostriatal glutamatergic transmission prevented alcohol withdrawal anxiety-like behavior. Fig. S9. Measurement of total distance and anxiety-like behavior of groups of mice as control in optogenetic LTP and LTD experiments. [file 12974_2022_2666_MOESM1_ESM.docx]

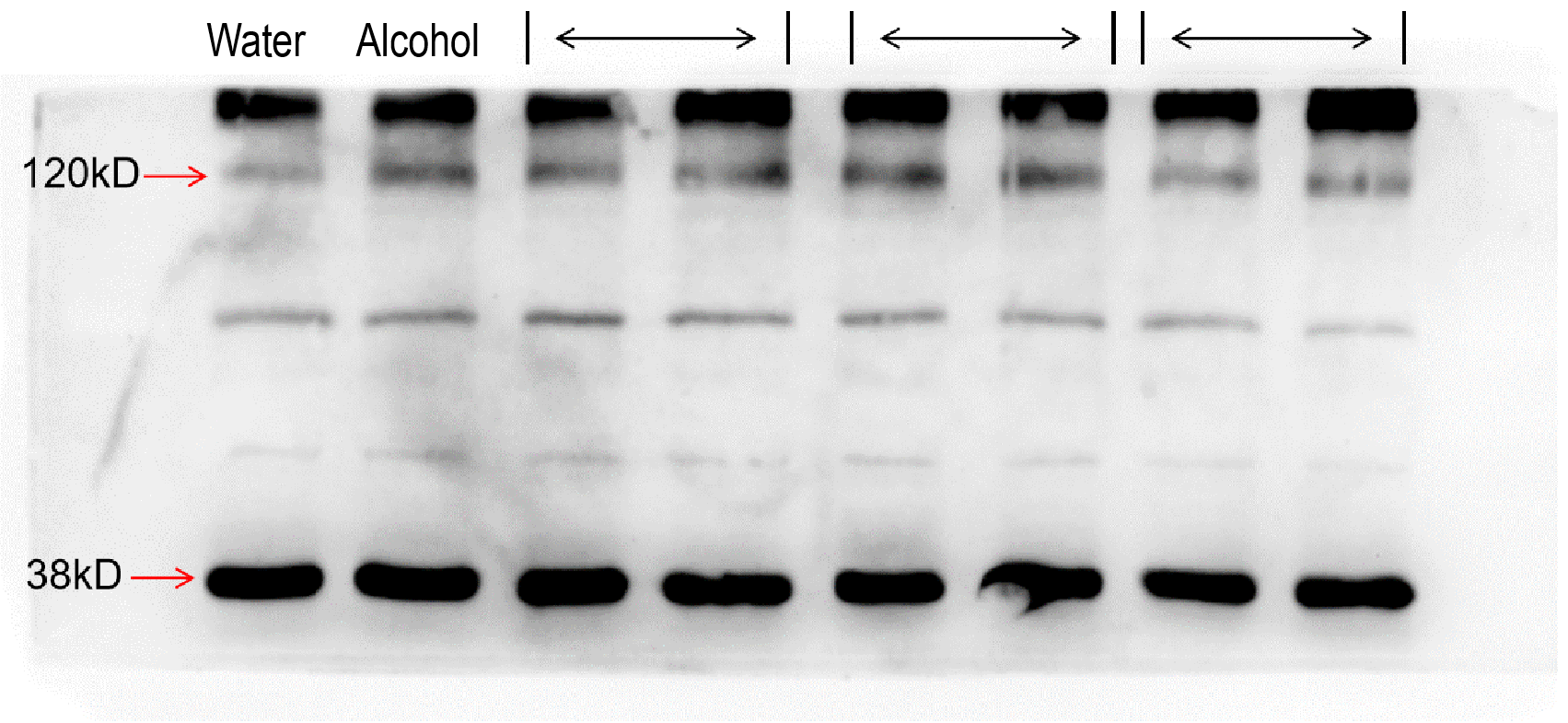


**1 2 3 4**


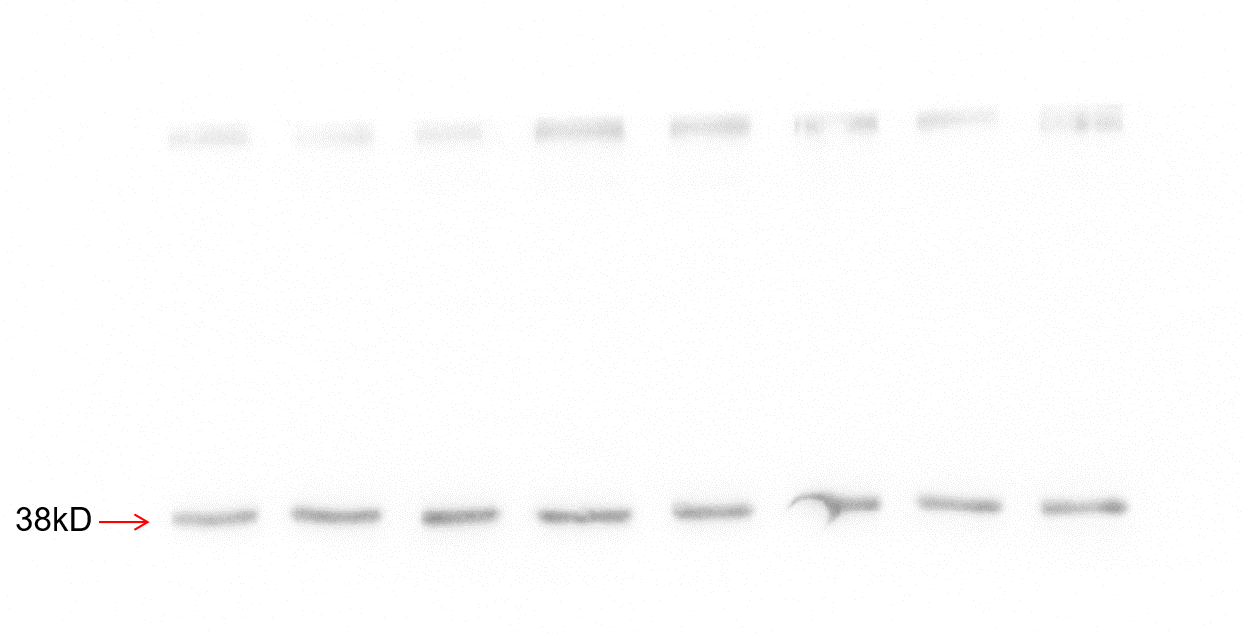


**Fig. S1 The expression of NLRP3 and GAPDH**. Gavage administration of C57BL/6 mice with 25% alcohol or water. 6h after the last operation, brain tissue from mice was taken and the expression of NLRP3 was measured by Western blotting. **(Up)** The expression of NLRP3 of water and alcohol group was showed in the unbroken PVDF membrane (120kD). **(Bottom)** The expression of GAPDH was used as a reference protein showing in the unbroken PVDF membrane (38kD).


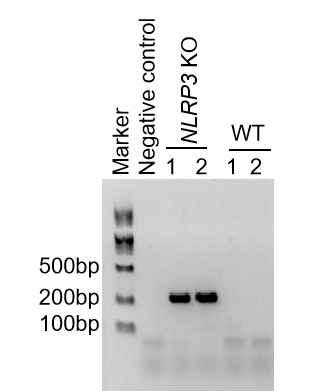


**Fig. S2** **Genotyping of NLRP3 KO mice.** DNA was extracted from tails and was as temple to performed PCR assay. The primers were designed to detect NLRP3 KO mice. The WT mice were added as control. The negative control is without DNA temple for PCR assay. The forward primer is located at the inserted neomycin cassette, the reverse one is at the gene body of NLRP3. The results showed that the product for PCR assay was only presented in NLRP3 KO mice.





**Fig. S3** **Measurement of total distance and anxiety-like behavior in experimental and control groups**. **A** No difference showed among WT group, WT+alcohol group and NLRP3 KO+alcohol group on the total distance travelled in the open field. *p* > 0.05, one-way ANOVA, n = 14 mice (WT), 12 mice (WT+Alcohol) and 12 mice (NLRP3 KO+Alcohol). **B** The total distance travelled in EPM suggested that there was no difference among WT group, WT+alcohol group and NLRP3 KO+alcohol group on locomotor performance. *p* > 0.05,one-way ANOVA. n = 8 mice (WT), 6 mice (WT+Alcohol) and 8 mice (NLRP3 KO+Alcohol). **C** Left, there was no difference between WT group and NLRP3 KO+Water group on the time spent in the centre area. *p* > 0.05, unpaired *t* test; Right, there was no difference between WT group and NLRP3 KO+Water group on the total distance traveled in the open field. *p* > 0.05, unpaired *t* test. n = 8 mice (WT) and 8 mice (NLRP3 KO+Water). **D** In EPM, no difference between WT group and NLRP3 KO+Water group on open-arm time (left), open-arm entries (middle) and total distance (right). *p* > 0.05, unpaired *t* test; n = 8 mice (WT) and 8 mice (NLRP3 KO+Water). Data are presented as mean±SEM.


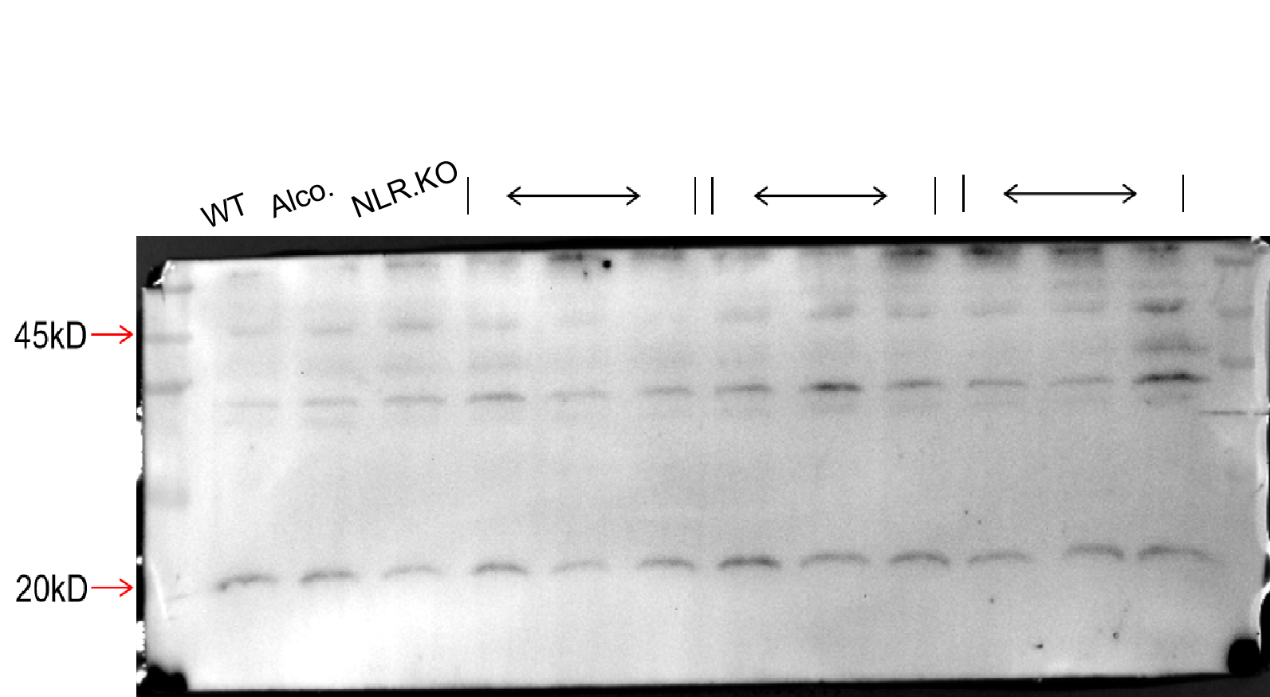


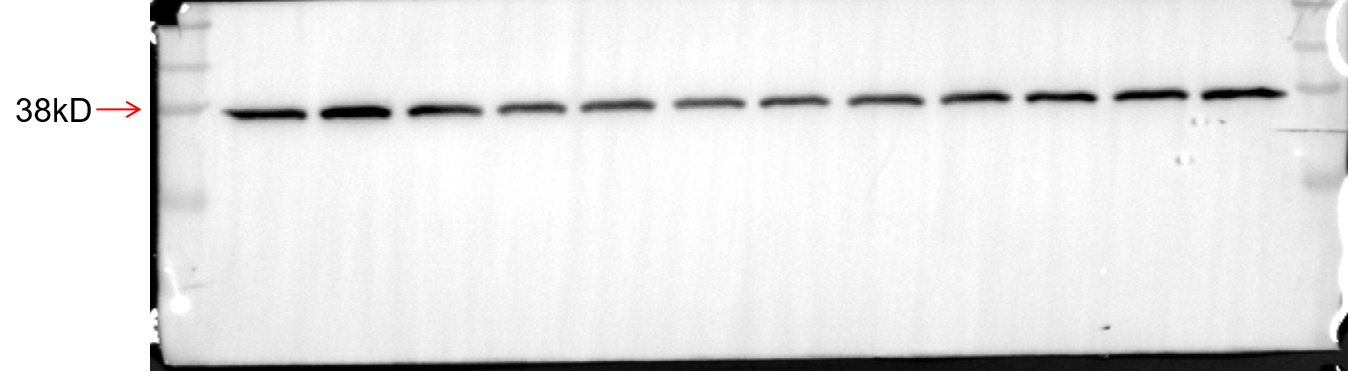


**Fig. S4** **The expression of caspase-1 and GAPDH in mPFC**. WT group, WT+alcohol group and NLRP3 KO+alcohol group mice were trained by binge drinking procedure as in Figure 2A. Tissues from the striatum were prepared after behavior test and the expression of caspase-1 and GAPDH was detected by Western blotting. **(Up)** The expression of pro-caspase-1 (45kD) and cleaved caspase-1 (20kD) of water and alcohol group was showed in the unbroken PVDF membrane. **(Bottom)** The expression of GAPDH was used as a reference protein showing in the unbroken PVDF membrane (38kD).


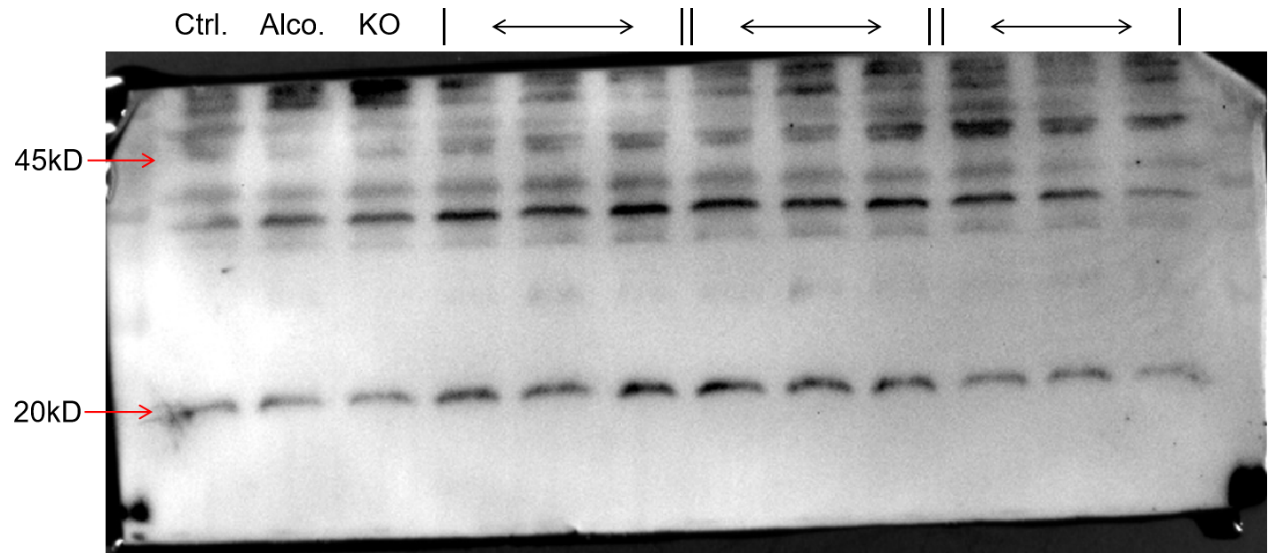


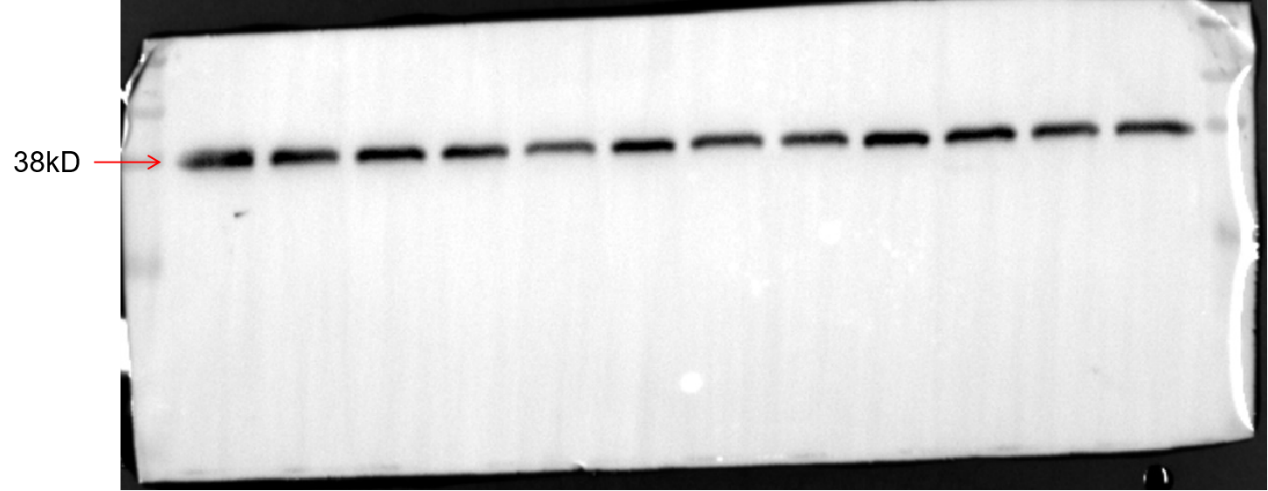


**Fig. S5** **The expression of Caspase-1 and GAPDH in striatum.** WT group, WT+alcohol group and NLRP3 KO+alcohol group were trained by binge drinking procedure as in Figure2A. Tissues from the striatum were prepared after behavior test and the expression of caspase-1 and GAPDH was detected by Western blotting. **(Up)** The expression of pro-caspase-1 (45kD) and cleaved caspase-1 (20kD) in striatum of water and alcohol group was showed in the unbroken PVDF membrane. **(Bottom)** The expression of GAPDH was used as a reference protein showing in the unbroken PVDF membrane (38kD).





**Fig. S6** **The identification of genotype difference between wild-tpye and NLPR3 KO control mice in Nissl staining and Western blotting tests. A** Sample images showing Nissl bodies in mPFC in WT group and NLRP3 KO+control group. scale bar = 50 μm. **B** Statistical analysis of nissl bodies in mPFC of two group mice. *p* > 0.05, unpaired *t* test, 3 mice from each group. **C** Sample images showing Nissl bodies in the striatum in WT group and NLRP3 KO+control group mice. scale bar = 50 μm. **D** Summary of the numbers of nissl bodies in striatum of two group mice. *p* > 0.05, unpaired *t* test, 3 mice from each group. **(E)** Left, the samples of Western blotting showing the expression of cleaved caspase-1 in WT group and NLRP3 KO+water group. Right, the relative expression of cleaved caspase-1 was lower in NLRP3 KO+control mice, **p* < 0.05, unpaired *t* test, n = 4 mice per group. **F (Up)** The expression of cleaved caspase-1 (20kD) of water and alcohol group was showed in the unbroken PVDF membrane. **(Bottom)** The expression of GAPDH was used as a reference protein showing in the unbroken PVDF membrane (38kD).





**Fig. S7** **The difference of PPR and NMDAR/AMPAR ratio between WT and NLRP3 KO control group. A** Sample traces showing paired-pulse ratios (PPR) from the two groups. **B** Data illustrated no difference of PPRs between the two groups*. p* > 0.05, unpaired *t* test, n = 9–19 neurons. **C** Sample traces of NMDAR/AMPAR ratio from the two groups. **D** Averaged data of NMDAR/AMPAR ratio showed no significant difference between the two groups. *p* > 0.05, unpaired *t* test, n = 8–16 neurons. WT groups were referenced from Figures 2D and 2E. Data are presented as mean±SEM.





**Fig. S8 Optogenetic induction of LTD in corticostriatal glutamatergic transmission prevented alcohol withdrawal anxiety-like behavior**. **A** Left, schematic diagram of open-field test after LTD induction. Right, low-frequency stimulation protocol is at 1 Hz and 900 pulses. **B** LTD induction in WT+alcohol+LTD group increased the time spent in the centre area compared to the WT+alcohol group. ***p* < 0.01, ****p* < 0.001 vs WT group, ^#^*p* < 0.05 vs WT+alcohol group, one-way ANOVA; n = 11 mice (WT), 6 mice (WT+Alcohol) and 6 mice (WT+Alcohol+LTD). **C** Left, sample traces of PPRs in striatal neurons of the three groups of mice. Right, calculation of the PPRs in the three groups of mice. **p* < 0.05, ^#^*p* < 0.05. n = 10-16 neurons from each group. **D** Left, sample traces of the NMDAR/AMPAR ratio in the three groups. Right, optogenetic induction of LTD decreased the NMDAR/AMPAR ratio compared to the WT+alcohol group. ****p* < 0.001, ^###^*p* < 0.001. n = 10-16 neurons from each group. **E** Left, sample traces of the AMPAR-EPSCs input-output curves in corticostriatal circuits in the three groups. Right, the amplitudes of AMPAR-EPSCs in WT+Alcohol+LTD group decreased compared to the WT+alcohol group. **p* < 0.05, ^#^*p* < 0.05. two-way RM ANOVA; n = 10-16 neurons from each group. Data are presented as mean±SEM.



 **Fig. S9** **Measurement of total distance and anxiety-like behavior of groups of mice as control in optogenetic LTP and LTD experiments. A** No difference showed on locomotor performance among the three groups in LTP expeiments (figure 5). *p* > 0.05, one-way ANOVA; n = 12 mice (WT+Alcohol), 11 mice (NLRP3 KO+Alcohol) and 11 mice (NLRP3 KO+Alcohol+LTP). **B** No difference showed on locomotor performance among the three groups in LTD experiments (fig. S8). *p* > 0.05, one-way ANOVA; n = 11 mice (WT), 6 mice (WT+Alcohol) and 6 mice (WT+Alcohol+LTD). **C-D,** AAV-tdTomato was as a control virus for LTP or LTD induction. **C** Left, NLRP3 KO+alcohol group and NLRP3 KO+alcohol+tdTomato+LTP group showed no difference on the time spent in the centre area. *p* > 0.05, unpaired *t* test. Right, the total distance travelled in open field showed that there was no difference between the two groups on locomotor performance. *p* > 0.05, unpaired *t* test, n = 11 mice (NLRP3 KO+Alcohol) and 8 mice (NLRP3 KO+Alcohol+tdTomato+LTP). **D** Left, WT+alcohol group and WT+alcohol+tdTomato+LTD group showed no difference on the time spent in the centre area. *p* > 0.05, unpaired *t* test. Right, no difference showed between the two groups on locomotor performance in open field. *p* > 0.05, unpaired *t* test, n = 6 mice (WT+Alcohol) and 7 mice (WT+Alcohol+tdTomato+LTD). Data are presented as mean±SEM.
